# Ligand-Dependent Optical Properties of Colloidal Ternary Spinel Oxide Nanocrystals Containing Transition Metals

**DOI:** 10.1021/acs.inorgchem.5c02179

**Published:** 2025-07-16

**Authors:** Revathy Rajan, Jordan C. Scalia, Luis R. De Jesús Báez, Kathryn E. Knowles

**Affiliations:** † Department of Chemistry, 6927 University of Rochester, Rochester, New York 14627, United States; ‡ Department of Chemistry, 12292 University at Buffalo − The State University of New York, Buffalo, New York 14260-3000, United States

## Abstract

Ternary spinel oxides of formula AB_2_O_4_ are semiconductors that possess compositionally and structurally tunable magnetic and optoelectronic properties that, when coupled with their extraordinary chemical and thermal stability, offer functional materials with applications in the fields of photocatalysis, solar energy conversion, gas sensing, and photoelectrochemistry. Nanocrystals of these materials offer the additional advantages of high surface area-to-volume ratios and the ability to use surface functionalization as a plausible strategy for tailoring their optoelectronic properties to improve their function in a specific application. Here, we demonstrate that surface-bound species can dominate the absorption spectra of colloidal ternary spinel oxide nanocrystals. We show that the surface functionalization of cobalt-containing systems with thiol ligands leads to the growth of an intense peak centered at 2.4 eV (518 nm) in their absorption spectra, which arises due to the formation of cobalt-thiolate linkages on the nanocrystal surface. We demonstrate that the observed optical change can be used to track ligand exchange reactions and assess the relative binding affinity of thiol, amine, and carboxylate ligands to the nanocrystal surface. This work highlights the significant role that surface chemistry can play in determining the optical properties of ternary spinel oxide nanocrystals.

## Graphical abstract

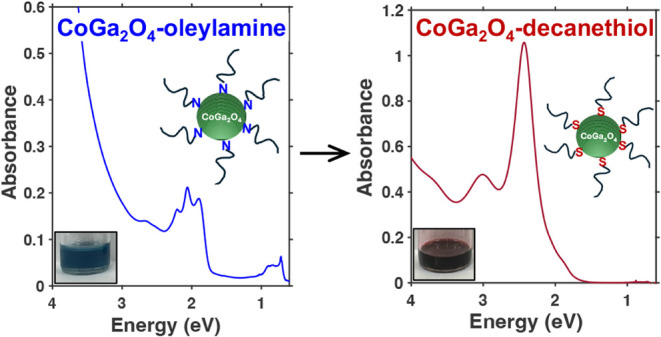

## Introduction

Ternary spinel oxides of general formula AB_2_O_4_ are attractive materials for applications ranging from pigments and catalysts to advanced energy materials due to their ability to accommodate multiple oxidation states and cation geometries within one material.^1,2^ The presence of both tetrahedral and octahedral sites within the spinel crystal structure offers unique opportunities for structural tunability through variations in the distribution of cations among these sites, which is parametrized by the inversion parameter.^2^ The redox flexibility of ternary spinel oxides containing transition metals that can adopt a variety of oxidation states is beneficial for applications in electro- or photocatalysis.^3–6^

In general, the catalytic performance of a colloidal nanocrystal is strongly influenced by its surface chemistry, including the availability of surface active sites;^7^ their electronic properties,^7,8^ coordination environment,^9,10^ and covalency;^11^ and the relative binding energies of ligands and substrates to the surface.^8^ Although there have been several detailed studies of the binding strength, surface density, and exchange chemistry of organic ligands on the surfaces of colloidal metal oxides containing main group or d^0^ metals, such as ZnO,^12–14^ HfO_2_,^15–17^ and ZrO_2_,^16,17^ there is a comparative lack of similar investigations into the behavior of ligands on the surfaces of metal oxide nanocrystals containing cations with partially filled d-shells (e.g., late transition metals such as Mn^2+/3+^, Fe^2+/3+^, Co^2+/3+^). Given their significant potential as photo- and electrocatalysts and their ability to accommodate a wide range of transition metals in various oxidation states and stoichiometries, ternary spinel oxides are an ideal platform to explore ligand chemistry on the surfaces of late transition metal oxide nanocrystals. However, determining the exact nature and impact of various surface species in ternary spinel oxide nanocrystals is remarkably challenging. For other colloidal nanocrystal systems, such as main group oxides or quantum-confined sulfide or selenide systems, nuclear magnetic resonance (NMR) and fluorescence measurements can be used to quantify ligand exchange reactions *in situ*.^18–20^ However, these techniques are not very compatible with materials containing transition metals that have unpaired electrons; this paramagnetism can lead to line broadening that complicates the use of NMR to quantify ligand binding.^21^ Furthermore, most transition metal oxides are not photoluminescent. Thus, there is a need for new methods to characterize ligand binding on the surfaces of transition metal oxide nanocrystals.

One alternative method that may be used to assess surface ligand binding is optical absorption spectroscopy. Prior examples of surface ligands impacting the optical absorption spectra of semiconductor nanocrystals have primarily focused on quantum-confined systems. Weiss and coworkers observed a bathochromic shift in the excitonic absorption peak (corresponding to a decrease in the optical band gap of ∼0.2 eV) upon addition of phenyldithiocarbamate (PTC) ligands to colloidal CdSe quantum dots (QDs).^22^ This shift is caused by leakage of the hole wave function into the ligand shell, and the magnitude of the shift increases with increasing quantum confinement.^23^ Kovalenko et al. observed a similar effect upon the addition of SnS_4_^4–^ to strongly confined PbS QDs.^24^ In systems that are not quantum confined like TiO_2_ thin films, Fujisawa and Hanaya have observed a bathochromic shift in the absorption onset upon functionalization with para-substituted benzoic acid derivatives, where the magnitude of the shift increases as the electron-donating ability of the benzoic acid derivative increases.^25^ Similar shifts have also been observed for ZnO films upon exposure to benzoic acid or benzenethiol derivatives.^25,26^ These shifts are attributed to interfacial ligand-to-metal charge transfer transitions between the highest occupied molecular orbital (HOMO) of the adsorbed benzoic acid ligands, the energy of which depends on the electron-donating ability of the substituent and the position of the conduction band minimum of the oxide film. Covalent bonding of organic ligands onto transition metal oxide nanocrystals, such as TiO_2_ or Fe_3_O_4_, and metallic nanocrystals, such as Ru, via linkages to alkynyl, aryl, or vinyllic carbons has also been demonstrated to enhance charge transfer at the nanoparticle/ligand interface.^27–29^

The work we report here extends the portfolio of ligand-induced optical changes in colloidal semiconductor nanocrystals to include colloidal ternary spinel oxides, which are not quantum confined. We chose CoGa_2_O_4_ (CGO) nanocrystals as a model system because its absorption spectrum contains isolated optical features that correspond to intra-atomic crystal field transitions within tetrahedral Co^2+^.^30,31^ Given the prominence of cobalt ligand-field transitions in the absorption spectra of colloidal CGO nanocrystals, we hypothesized that changes in the coordination environment of cobalt at the surface could impact the optical spectra of these nanocrystals. Furthermore, among ternary spinel oxides, materials containing cobalt have gained significant attention in recent years. In catalysis, they are used in oxygen evolution^32,33^ and reduction^34,35^ reactions and as photocatalysts for pollutant and dye degradation.^5,36^ The ability to tune the properties of CGO nanocrystals through surface manipulation could also offer exciting possibilities for tailoring its performance to specific optoelectronic applications.

Here, we demonstrate that surface ligands can induce an optical response upon binding to specific transition metals on the surface of a nanocrystal. Unlike prior examples involving quantum confined nanocrystals, where shifts in the band gap were observed upon changes in surface ligands, here we observe the development of an entirely new absorption feature. Surface functionalization of CGO nanocrystals with thiol ligands leads to the growth of a peak centered at 2.4 eV (518 nm) in their absorption spectra. Upon displacement of the thiol ligands with oleic acid, this peak disappears, confirming its association with the formation of cobalt-thiolate linkages on the nanocrystal surface. Similar changes are also observed in other ternary spinel oxide nanocrystals containing cobalt or nickel; both metals form coordination complexes with thiolates that contain metal-ligand charge transfer features.^37–40^ We therefore propose that the 2.4 eV feature corresponds to a localized ligand-to-metal charge transfer (LMCT) transition within surface-bound cobalt-thiolate complexes. We demonstrate that, by using this feature as a spectroscopic handle on the composition of the surface ligands, we can qualitatively assess the relative binding energies of 1-decanethiol, oleic acid, and oleylamine ligands to CGO surfaces.

## Experimental Methods

### Materials

The following chemicals were purchased commercially and were used as received without further purification: gallium­(III) acetylacetonate (99.99+% Ga, Strem Chemicals, Inc.), iron­(III) acetylacetonate (99%, Strem Chemicals, Inc.), cobalt­(II) acetylacetonate (99%, Thermo Scientific), cobalt­(III) acetylacetonate (98+%, Strem Chemicals, Inc.), nickel­(II) acetylacetonate (95%, Sigma-Aldrich), zinc­(II) acetylacetonate hydrate (98%, Strem Chemicals, Inc.), oleylamine (≥98%, Sigma-Aldrich), oleic acid (90%, Sigma-Aldrich), dibenzyl ether (98+%, Thermo Scientific), ethanol (200 proof pure, Koptec), hexane (Fisher Chemical), toluene (VWR Chemicals), tetrachloroethylene (99%, Thermo Scientific), methanol (Fisher Chemical), acetone (Fisher Chemical), tetrahydrofuran (Fisher Chemical), 3-mercaptopropionic acid (≥99%, Sigma-Aldrich), mercaptosuccinic acid (97%, Sigma-Aldrich), decanethiol (96%, Thermo Scientific), 1-octadecene (90%, Sigma-Aldrich), CDCl_3_ (99.8%, Cambridge Isotope Laboratories, Inc.), nitric acid (Trace Metal grade, Fisher Chemical), hydrochloric acid (certified ACS Plus, Fisher Chemical), L-malic acid (Oakwood Chemical), propionic acid (≥99.5%, Sigma-Aldrich), dibutyl sulfide (≥98%, Sigma-Aldrich). Succinic acid was recrystallized from acetone. Cole-Parmer Essentials PTFE Chromatography Syringe Filters with 0.2 μm pore size and 25 mm diameter were used to filter the samples.

### Synthesis of MGa_2_O_4_ and MFe_2_O_4_ Nanocrystals

Gallium­(III) acetylacetonate (0.7 mmol, 0.257 g), metal­(II) acetylacetonate (0.35 mmol), oleylamine (2.7 mmol, 0.722 g), oleic acid (2.7 mmol, 0.762 g), and benzyl ether (10 mL) were added to a 25 mL Teflon insert. This mixture was then stirred for 10 min to make a suspension. The Teflon insert was placed inside a sealed stainless-steel autoclave and heated at 230 °C. After 24 h, the autoclave was allowed to cool down to room temperature over the course of 18 to 24 h. Three cycles of precipitation with ethanol followed by centrifugation were performed to clean the nanocrystals. The supernatant was discarded, and the precipitate was dispersed in hexane to obtain colloidal suspensions whose colors depend on the identity of M^2+^: blue for M^2+^ = Co, mint green for M^2+^ = Ni, and colorless for M^2+^ = Zn. The same procedure was followed for the synthesis of γ-Ga_2_O_3_, except for omitting the addition of metal­(II) acetylacetonate. The final suspension of γ-Ga_2_O_3_ nanocrystals is colorless. For the synthesis of ferrites (MFe_2_O_4_ nanocrystals), iron­(III) acetylacetonate (0.7 mmol, 0.2472 g) was used instead of gallium­(III) acetylacetonate. All the ferrite nanocrystals had shades of yellowish brown. Detailed structural characterization of all materials other than CGO can be found in Figures S1 and S2.

### Ligand Exchange Procedures

#### CGO-OAm-DT, CGO-OA-DT

The ligand exchange procedures were conducted in tetrachloroethylene to avoid near-infrared solvent absorption features. To 10 mL of 1 mg/mL CGO suspension in tetrachloroethylene, 50 μL of oleylamine (OAm, 0.152 mmol) or oleic acid (OA, 0.157 mmol) was added to obtain a colloidal suspension of oleylamine-coated CGO (CGO-OAm) or oleic acid-coated CGO (CGO-OA) nanocrystals. After shaking and sonication, the colloidal dispersion was passed through a PTFE syringe filter (0.2 μm pore diameter) to obtain an optically clear solution. To this colloidally stable blue solution, 60 μL (approximately 1:10 molar ratio of Co to thiol based on the per cobalt molar extinction coefficient of CGO nanocrystals, see Table S2 and Figure S3 for details) of 1-decanethiol (DT) was added and the dispersion was shaken well. The blue solution changed to red gradually with time. The observed optical changes were monitored using UV-vis absorption spectroscopy. The nanocrystals obtained from the thiol ligand exchange of CGO-OAm are labeled CGO-OAm-DT and the nanocrystals obtained from the thiol ligand exchange of CGO-OA are labeled CGO-OA-DT.

#### CGO-MSA, CGO-MPA

These ligand exchange reactions were conducted using a modification of a published method.^41,42^ To 2 mL of a 10 mg/mL suspension of CGO-OAm in hexane, 76 mg of mercaptosuccinic acid (MSA) or 44 μL of mercaptopropionic acid (MPA) (approximately 1:10 molar ratio of Co to thiol) was added. One mL of a potassium phosphate buffer of concentration 0.1 M at a pH of 8.8 and 1 mL of chloroform were added. This mixture was sonicated for an hour and later centrifuged and washed with ethanol. The dry precipitate after washing was dispersed in 2 mL water and sonicated to obtain the CGO-MSA and CGO-MPA nanocrystal solution. Absorption spectra of 20× diluted solutions of CGO-MSA and CGO-MPA were collected after passing through a 0.2 μm syringe filter and these absorbance values were then multiplied by 20 to obtain the spectra plotted in Figure 2B.

#### CGO-DT, NGO-DT, ZGO-DT, γ-Ga_2_O_3_-DT

To 2 mL of a 10 mg/mL suspension of CoGa_2_O_4_ (CGO), NiGa_2_O_4_ (NGO), ZnGa_2_O_4_ (ZGO) or γ-Ga_2_O_3_ nanocrystals in hexane, 20 μL of oleylamine was added and sonicated. After filtration, 105 μL of DT was added to each of these suspensions. This amount of ligand corresponds to approximately a 1:10 molar ratio of Co:thiol for the CGO sample. A gradual color change to red was observed over time for CGO-DT and NGO-DT but not for ZGO-DT and γ-Ga_2_O_3_-DT. The observed optical changes were monitored using UV-vis absorption spectroscopy.

#### CFO-DT, NFO-DT, ZFO-DT, Fe_3_O_4_-DT

To 2 mL of a 0.1 mg/mL suspension of CoFe_2_O_4_ (CFO), NiFe_2_O_4_ (NFO), ZnFe_2_O_4_ (ZFO) or Fe_3_O_4_ nanocrystals in hexane, 20 μL of oleylamine was added and sonicated. After filtration, 11 μL of DT was added to each of these dispersions. This amount of thiol corresponds to a molar ratio of approximately 1:100 Co:thiol for CFO. A gradual change in color from light brown to dark red was observed for CFO-DT only. NFO-DT, ZFO-DT, and Fe_3_O_4_-DT did not show any detectable changes in their UV–vis absorption spectra.

### Reversibility Experiment

After washing once with methanol, CGO-OAm-DT nanocrystals were resuspended in toluene to obtain 5 mL of 0.5 mg/mL colloidal dispersion in a 25 mL 2-neck round bottom flask. A water-cooled Liebig condenser was attached to one neck of the flask and the other side of the condenser was connected to a Schlenk line. After three cycles of evacuation followed by backfilling with nitrogen at room temperature, the solution was continuously stirred and heated to reflux at 115 °C in a silicone oil bath. After about 30 min of heating, 0.5 mL of oleic acid was added to the reaction. The red reaction mixture changed color to pale yellow gradually after 24 h. Upon addition of methanol to the reaction mixture followed by centrifugation, a pale blue precipitate was recovered. For the control reversibility experiment, the procedure remained the same except for the omission of the addition of oleic acid.

### Nanomaterials Characterization

#### Powder X-ray Diffraction

We measured powder X-ray diffraction on dried powders of the nanocrystals using a Rigaku XtaLAB Dualflex Synergy-S diffraction system with Mo Kα radiation (λ = 0.71073 Å). The 2θ values obtained using the Mo source were then converted to 2θ values corresponding to the wavelength of a Cu Kα source (λ = 1.54148 Å) following previously reported methods to compare our obtained spectra to standard data in the JCPDS databases.^43,44^

#### X-ray Photoelectron Spectroscopy (XPS)

XPS measurements were performed on three separate spots of each sample to ensure data reproducibility. All the samples were prepared at room temperature by drop-casting a nanocrystal suspension in hexane onto a cleaned Si wafer. These Si wafers were then electrically grounded to the sample bar with carbon tape. The XPS measurements were recorded with a Kratos Axis Ultra DLD system equipped with a monochromatic Al Kα (*h*ν = 1486.6 eV) X-ray source. During the measurements, pressure in the main chamber was kept below 5 × 10^–7^ mbar. Charge compensation was carried out via a neutralizer running at a current of 1.9 × 10^–6^ A, a charge balance of 2.6 eV, and a filament bias of 1.3 V. The X-ray gun was set to 10 mA emission. Binding energies were referenced to the C 1s peak arising from adventitious carbon with a binding energy of 284.8 eV. The C 1s, O 1s, S 2p, S 2s, Ga 2p, Ga 3d, and Co 2p core levels were recorded with a pass energy of 20 eV. We collected three scans for carbon, gallium, and oxygen, five scans for cobalt, and seven scans for sulfur. CasaXPS (version 2.3.22PR1.0.) was used to perform the XPS data analysis. The Shirley function was used for background subtraction, and the XPS signals were fitted using the GL(30) symmetric line shape (30% Lorentzian and 70% Gaussian)^45,46^ with the CasaXPS Component Fitting tool.

#### Transmission Electron Microscopy (TEM)

TEM micrographs were obtained using a FEI Tecnai F20 transmission electron microscope with a beam energy of 200 kV. The nanocrystal samples were drop-cast from hexane onto copper grids coated with lacy carbon. The diameter of the particles was measured using ImageJ software (version 1.52a).^47^

#### ^1^H NMR Spectroscopy

^1^H NMR spectra were collected on a JEOL 400 MHz spectrometer. All samples were dispersed in CDCl_3_.

#### Optical Absorption Spectroscopy

Optical absorption spectra of colloidal dispersions of nanocrystals were collected in a cuvette with a path length of 2 mm using an Agilent Cary 7000 UV–Vis spectrometer.

#### X-ray Absorption near-Edge Spectroscopy (XANES)

All XANES spectra were collected utilizing an easyXAFS 300+ spectrometer. Samples were prepared for data collection by using cellulose as a binding agent, homogenized with a mortar and pestle, pressed into a thin 13 mm diameter pellet using a Carver hydraulic press, and sealed between two pieces of Kapton tape. The data collection was performed at an operating voltage and current of 30 kV and 20 mA, respectively, using Si (5,3,1) crystal analyzer as monochromator. One scan was obtained for each standard, while two separate scans were collected for the experimental samples in transmission mode at the cobalt K-edge at room temperature. Energy correction was carried out using the difference in energy position between the collected samples and the position of the first inflection point on the first derivative of the metal foil standard. Normalization was performed using the ATHENA suite.

## Results and Discussion

### Surface Functionalization of Cobalt-Containing Systems with Thiol Ligands Gives Rise to Intense New Absorption Features

Most previously reported syntheses of CoGa_2_O_4_ rely on high-temperature solid-state reactions,^48^ electrospinning-calcination processes,^49^ supercritical hydrothermal synthesis,^50^ or complex sol–gel processes.^51^ These methods often suffer from high energy consumption and limited control over particle morphology. In contrast, we employ a solvothermal synthesis method using a mixture of metal acetylacetonate salts in benzyl ether in the presence of oleic acid and oleylamine (Figure 1A). This method of synthesis is a modification of a procedure that has already been reported to access spinel ferrite nanocrystals (MFe_2_O_4_, M = Co, Ni, Zn),^43,52^ and is similar to a previously reported heat-up synthesis conducted at ambient pressure that included 1,2-hexadecanediol in the reaction mixture.^5^ Our approach enables the synthesis of CGO nanocrystals at relatively low temperatures (∼230 °C compared to 400–800 °C in the other synthesis methods). Furthermore, the use of oleic acid^53,54^ and oleyl amine^53,55^ enables access to colloidally stable nanocrystal samples. Compared to the previously reported solution-phase synthesis, our elimination of 1,2-hexadecanediol, extension of the reaction time to 24 h, and use of an autoclave produce larger nanocrystals (d = 8.6 ± 2.2 nm compared to d ∼ 5 nm for the previously reported nanocrystals).^5^ We also demonstrate that this solvothermal method can be extended to the synthesis of other ternary spinel gallium oxide nanocrystals, such as NiGa_2_O_4_ (NGO) and ZnGa_2_O_4_ (ZGO, see Figure S1), offering a general strategy for the controlled synthesis of colloidal ternary spinel oxide materials. To improve the colloidal stability of the as-synthesized nanocrystals, they are treated with a small amount of oleic acid (OA) or oleylamine (OAm) after the synthesis to make an oleic acid-coated or an oleylamine-coated nanocrystal, respectively.

**Figure 1:**
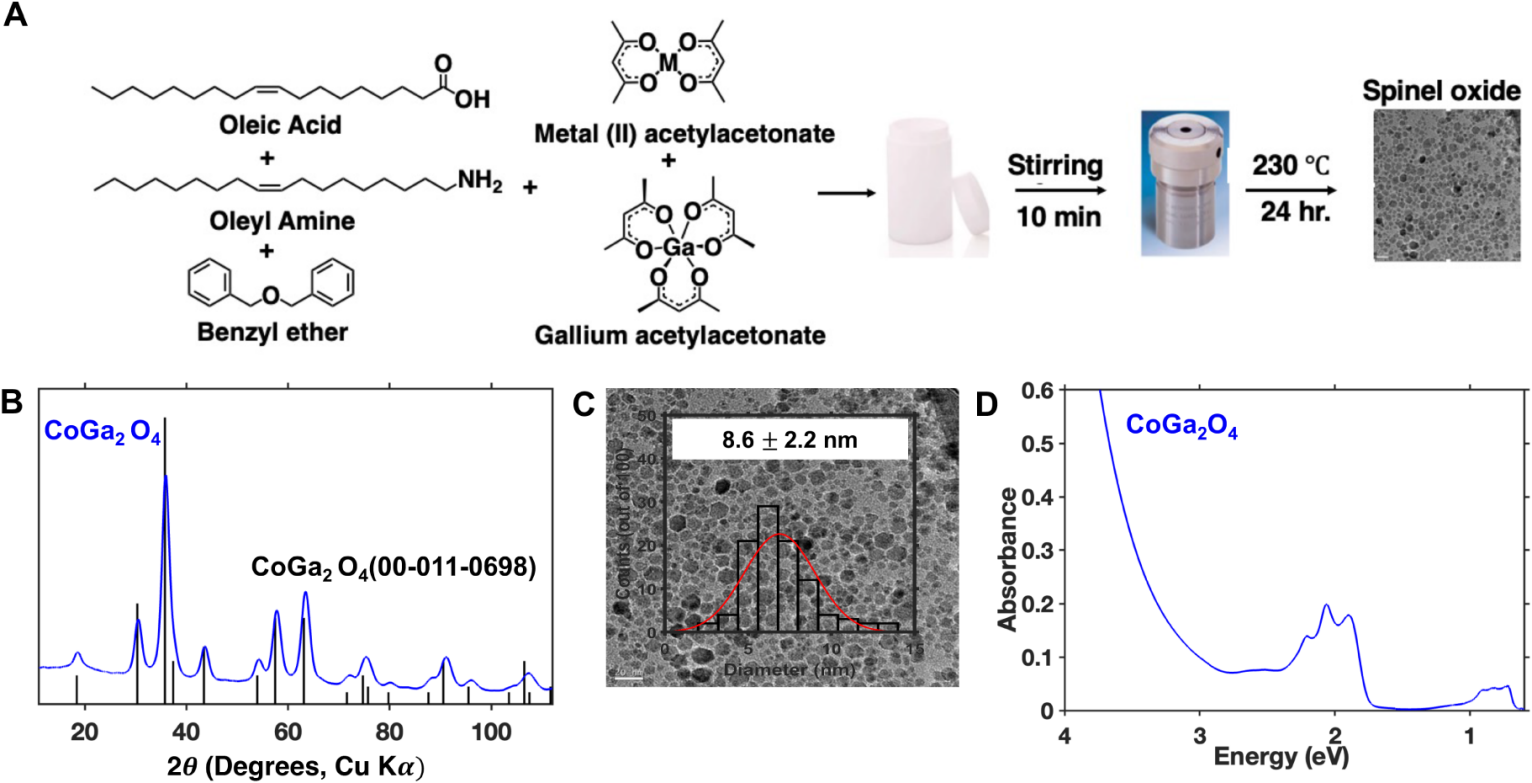
(A) Illustration of the solvothermal method for synthesizing spinel gallium oxides (CGO, NGO, ZGO). (B–D) Powder XRD pattern (B), TEM image with size distribution (C), and optical absorption spectra of a colloidal dispersion in tetrachloroethylene (D) of the as-synthesized CoGa_2_O_4_ nanocrystals.

All CGO nanocrystals synthesized by this method exhibit a phase-pure spinel structure by powder X-ray diffraction and are roughly spherical with diameters between 6 and 9 nm (Figure 1B,C). High-resolution TEM images reveal single-crystalline particles with visible lattice fringes corresponding to *d*-spacings that are consistent with the spinel structure (see Figure S4). We note that the powder X-ray diffraction pattern shown in Figure 1B for our CGO nanocrystals corresponds to a lattice parameter of 8.272 ± 0.003 Å (see Table S1) whereas the lattice parameter for the standard sample (JCPDS 00-011-0698) is 8.323 Å. Analysis of the composition of our CGO nanocrystals by inductively coupled plasma-mass spectrometry (ICP-MS) yields a nonstoichiometric Co:Ga ratio of 0.7:2, indicating that there is a cobalt deficiency in these nanocrystals. Given that the lattice parameter of γ-Ga_2_O_3_ is 8.238 Å,^56^ which is smaller than that of CoGa_2_O_4_, we attribute the observed lattice contraction in our CGO nanocrystals to this cobalt deficiency. The UV-vis absorption spectrum of a suspension of CGO nanocrystals in tetrachloroethylene contains two sets of isolated features spanning 1.8–2.3 eV and 0.8–1 eV that are consistent with crystal field transitions within tetrahedral Co^2+^ (Figure 1D).^30,31^ The average per-cobalt extinction coefficient (ε) of CGO nanocrystals at 2.06 eV was found to be 45 ± 4 M^–1^ cm^–1^ (see Table S2 and Figure S3). We used this extinction coefficient to estimate the stoichiometry of the ligand exchange reactions described below.

Upon addition of thiol ligands, namely mercaptosuccinic acid (MSA), mercaptopropionic acid (MPA), and 1-decanethiol (DT) (Figure 2A), to a colloidal dispersion of oleylamine-coated CGO (CGO-OAm) nanocrystals, we observe a color change from blue to reddish brown (Figure 2C). This color change was accompanied by the development of intense new absorption features around 2.4 and 3 eV (Figure 2B). Upon binding nanocrystal surfaces, MPA and MSA are known to provide a hydrophilic exterior that enables aqueous dispersibility.^57^ Addition of water to CGO-MSA and CGO-MPA samples produced turbid dispersions (Figure 2C). Optical absorption spectra were collected after diluting these dispersions by a factor of 20 and passing these dispersions through a PTFE syringe filter (<0.2 μm) in an effort to remove aggregates and minimize optical scattering; however we were unable to completely eliminate the scattering background (Figure 2B). In contrast, the hydrophobic tail of decanethiol preserves the dispersibility of CGO nanocrystals in nonpolar organic solvents.

**Figure 2:**
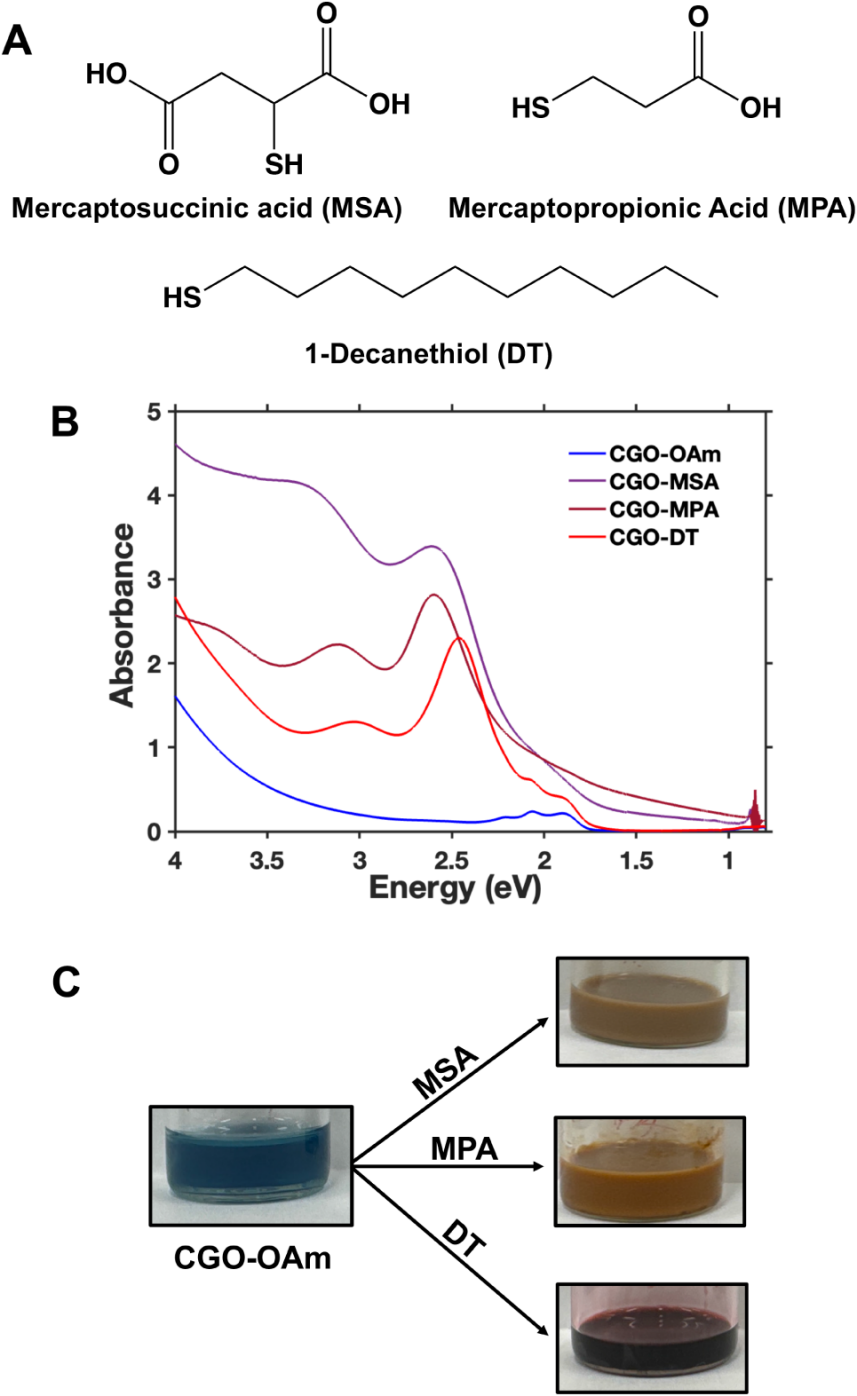
(A) Structures of different thiol ligands. (B) UV-vis spectra of CGO-OAm before and after the addition of thiol ligands (1:10 Co:thiol ratio). Blue: As-synthesized CGO capped with oleylamine in hexane (CGO-OAm), Purple: CGO after addition of mercaptosuccinic acid and redispersion in water (CGO-MSA), Maroon: CGO after addition of mercaptopropionic acid and redispersion in water (CGO-MPA), Red: CGO after addition of 1-decanethiol in hexane (CGO-DT). The CGO-MSA and CGO-MPA dispersions were diluted by a factor of 20 and passed through a syringe filter prior to data collection; the spectra shown here were thus multiplied by a factor of 20 to account for this dilution. (C) Pictures of CGO nanocrystals showing the color change from blue (before thiol ligand addition) to reddish brown (after thiol ligand addition).

To determine the origin of this color change and the corresponding strong absorption features, we examined the impact of DT addition on other ternary spinel gallium oxide and spinel ferrite nanocrystals. NGO, which was originally mint-green in color, changed to red gradually and developed absorption peaks centered at 2.4, 3, and 3.7 eV upon addition of 1-decanethiol (Figure 3A). In contrast to CGO-DT, for which the most intense peak is the one centered at 2.4 eV, for NGO-DT, the 3.7 eV peak is the most intense. We also note that, unlike CGO, NGO did not exhibit any optical response to the addition of MSA and MPA (see Figure S5). ZGO and γ-Ga_2_O_3_ both remained colorless before and after addition of DT and did not exhibit any color changes. When thiol ligands were added to spinel ferrite nanocrystals such as Fe_3_O_4_, CoFe_2_O_4_ (CFO), NiFe_2_O_4_ (NFO), and ZnFe_2_O_4_ (ZFO), only CGO showed significant spectral changes (Figure 3B). To further investigate these findings, we performed ligand exchanges on the CGO and NGO with their nonthiol (alcoholic) counterparts, namely malic acid (MA), succinic acid (SA), and propionic acid (PA) (Figure S6). Interestingly, no spectral changes were observed upon addition of these alcoholic ligands. Notably, the solutions became turbid, suggesting that ligand exchange did indeed occur.

**Figure 3:**
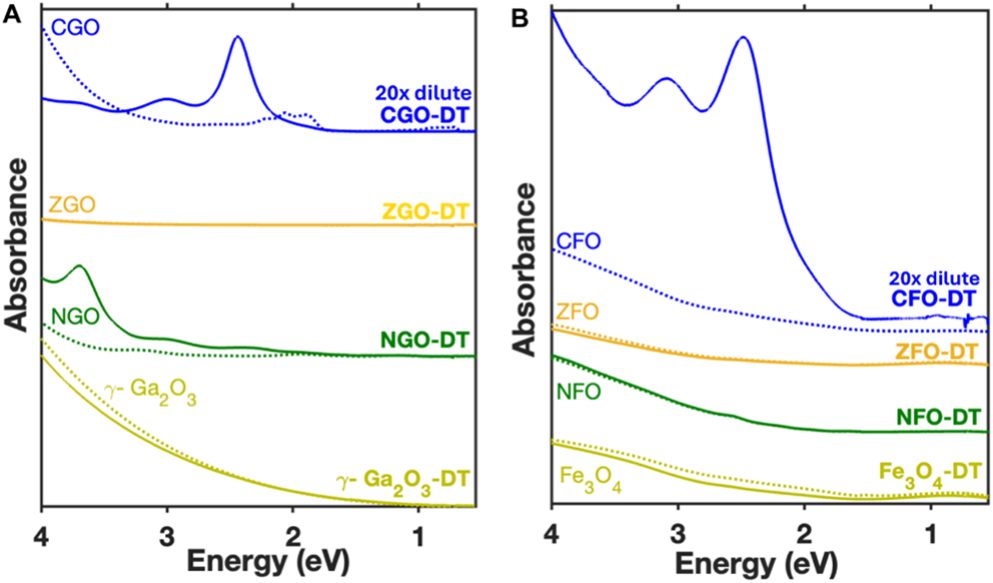
(A) Absorption spectra of oleylamine-coated spinel gallium oxides before (dotted lines) and after (solid lines) addition of decanethiol (Co:DT = 1:10, concentration of nanocrystals is 10 mg/mL). (B) Absorption spectra of ferrites before and after addition of decanethiol (Co:DT = 1:100, concentration of nanocrystals is 0.1 mg/mL).

Given our suspicion that binding of the thiol ligands to the nanocrystal surface is responsible for the observed color change, we investigated how changes to the identity of the initial surface ligands impact the rate and extent of the observed spectral changes using CGO as our model system. We varied the initial surface ligands by adding oleylamine or oleic acid to the purified nanocrystals prior to addition of 1-decanethiol (see Figure S7 for characterization of the differences in the surface ligand chemistry of CGO-OAm and CGO-OA). We observe a larger magnitude of spectral changes when we start with oleylamine-capped nanocrystals (CGO-OAm) compared to oleate-capped nanocrystals (CGO-OA) (Figure 4). We attribute this difference to a difference in the binding affinity of oleylamine and oleate ligands to the nanocrystal surface: oleylamine tends to be the weaker ligand.^58^ Figure 4A indicates that the ligand exchange reaction approaches equilibrium after approximately 48 h at room temperature without stirring for both CGO-OAm and CGO-OA, however, the final intensity of the peak at 2.4 eV is >4 times larger for the oleylamine-capped nanocrystals than for the oleate-capped nanocrystals. These data suggest that the thiol ligands can more efficiently displace oleylamine than oleate.

**Figure 4:**
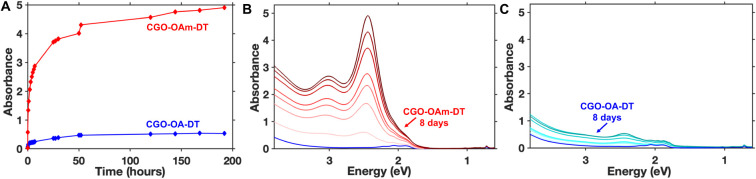
(A) Plot of absorbance (at 2.4 eV) versus time showing the impact of different initial surface ligands, oleic acid (blue) and oleylamine (red), on the rate and extent of the spectral changes observed after addition of 1-decanethiol. Absorption spectra collected of CGO-OAm-DT (B) and CGO-OA-DT (C) at various time points after 1-decanethiol addition spanning a period of 8 days.

### Characterization of Surface Species Formed Upon Addition of Decanethiol to CGO

We hypothesize that the massive optical change we observe upon addition of 1-decanethiol to CGO-OAm is due to a successful ligand exchange from oleylamine to 1-decanethiol on the surface of the CGO nanocrystals. Raman spectra of CGO-OAm-DT exhibit features consistent with the formation of cobalt-sulfur bonds (see Figure S8). Addition of a thioether to CGO-OAm does not induce any changes in the absorption spectra, suggesting that 1-decanethiol binding to cobalt sites as 1-decanethiolate is responsible for the observed spectral changes (see Figure S9). Addition of methanol to CGO-OAm-DT followed by centrifugation produces a dark red pellet and a colorless supernatant. Characterization of the dark red pellet by powder XRD and TEM reveals that it contains phase-pure spinel nanocrystals with morphologies that are nearly identical to the as-synthesized CGO-OAm sample (see Figure S10). These observations suggest that the dark red species formed upon addition of 1-decanethiol to CGO-OAm is bound to the surface of the CGO nanocrystals.

To further test the hypothesis that the observed spectral changes arise from surface-bound cobalt-thiolate complexes, we measured ^1^H NMR spectra of the initial oleylamine-capped nanocrystals (CGO-OAm) and the 1-decanethiol-exchanged (CGO-OAm-DT) samples. We characterized CGO-OAm-DT both immediately following ligand exchange (CGO-OAm-DT unwashed) and after performing one methanol wash (CGO-OAm-DT washed) to remove unbound ligands. Figure 5 compares these spectra with the ^1^H NMR spectra of pure oleylamine and 1-decanethiol. Compared to the free ligands, line broadening was visible in the ^1^H NMR spectra of both the pre- and post-DT ligand-exchanged nanocrystals (Figure 5A). This broadening could be due to the faster T_2_ relaxation caused by the reduced rotational mobility of the bound ligands^59^ (oleylamine in the pre-ligand-exchange samples and 1-decanethiol in the post-ligand-exchange samples). The broadening that we observe could also be due to the presence of paramagnetic cobalt ions in our nanocrystals,^60^ thus complicating the ability of ^1^H NMR spectra to quantify free and bound ligands.

Nevertheless, these ^1^H NMR spectra reveal some informative qualitative observations. The peak at 5.3 ppm, which corresponds to the vinyllic protons of oleylamine,^61^ is present in the CGO-OAm-DT unwashed, postligand exchanged sample and absent in the sample of CGO-OAm-DT washed with methanol. This observation indicates the presence of free oleylamine ligands that are removed by the methanol wash. The ^1^H NMR spectrum of 1-decanethiol (Figure 5A) is characterized by several distinct signals.^62,63^ The peak around 1.31 ppm corresponds to the thiol proton (−S**H**).^63^ A quartet near 2.51 ppm arises from the methylene group directly adjacent to the thiol group (the α-protons, −C**H**_**2**_–SH). A complex multiplet between 1.2 and 1.6 ppm represents the signals from the remaining methylene groups along the carbon chain. Finally, a triplet around 0.8–0.9 ppm is observed for the terminal methyl group (−C**H**_3_). The 1.31 ppm peak due to the thiol protons was present in free decanethiol and in the sample measured immediately after ligand exchange, but prior to the methanol wash (CGO-OAm-DT unwashed). We suspect this peak arises from the presence of free 1-decanethiol in the unwashed sample. The presence of the 2.51 ppm quartet corresponding to the α-protons in the post-ligand-exchange pre-wash sample is also an indication of the presence of free 1-decanethiol. These peaks at 1.31 and 2.51 ppm are both absent in the exchanged samples after the methanol wash (CGO-OAm-DT washed), indicating that free 1-decanethiol was removed upon washing with methanol. Broadening in the peaks, together with the absence of the thiol protons, suggests the presence of thiolate ligands bound to the nanocrystal surface in the post-ligand-exchanged sample. Notably, the multiplets at both ∼5.3 ppm and ∼2.1 ppm, which corresponds to −C**H**=C**H**– and −C**H**_2_– adjacent to −C**H**=C**H**– protons of oleylamine,^64^ are completely absent in the washed, exchanged sample (CGO-OAm-DT-washed), consistent with replacement of oleylamine ligands with 1-decanethiol ligands. The disappearance of the oleylamine signals also rules out formation of stable surface-bound alkylammonium/thiolate ion pairs analogous to the alkylammonium/carboxylate pairs reported by Owen and coworkers.^65^ ICP-MS analysis of a washed CGO-OAm-DT sample revealed a S:Co:Ga ratio of 0.07:0.7:2 (see Supporting Information). The Co:Ga ratio of 0.7:2 is the same as that observed for CGO nanocrystals prior to ligand exchange, which indicates that the 1-decanethiol ligands do not leach cobalt out of the nanocrystals and is consistent with our conclusion that the cobalt-thiolate species remain bound to the nanocrystal surface. The S:Ga ratio of 0.07:2 corresponds to a density of surface-bound thiols of 1.4 ± 0.4 nm^–2^, where the estimated uncertainty arises primarily from the heterogeneity of the nanocrystal size (see Supporting Information for details). This surface density is comparable to the surface densities of primary amines or amino acid ligands reported for metal oxide nanocrystals such as ZnO, HfO_2_, and Fe_3_O_4_.^14,66,67^ We note that the data presented in Figure 3 indicate that only the DT ligands bound to surface cobalt sites contribute to the optical feature observed at 2.4 eV; however, given the ternary and nonstoichiometric composition of our nanocrystals, we cannot ascertain from these data what fraction of the bound 1-decanethiol ligands participate in this absorption process.

**Figure 5:**
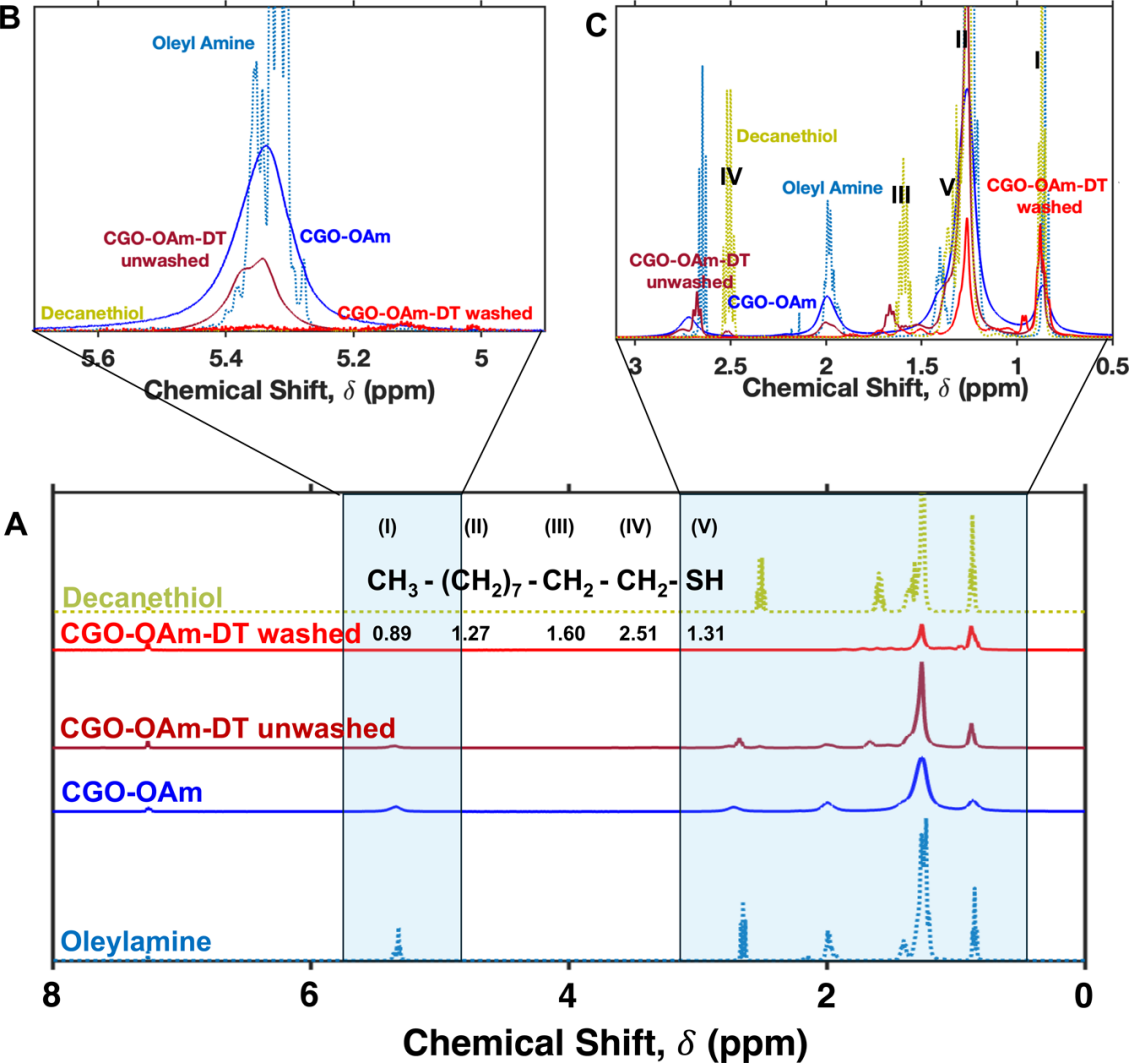
(A) Stacked ^1^H NMR spectra of CGO-OAm before and after adding 1-decanethiol and subsequently washing with methanol along with the ^1^H NMR spectra of free OAm and free DT. (B,C) Zoomed in ^1^H NMR spectra of the regions highlighted in part A, corresponding to vinyllic (B) and aliphatic (C) spectral regions.

Although our data suggest that 1-decanethiol binds to CGO nanocrystals in the form of 1-decanethiolate, the fate of the thiol proton remains unclear. Thiols have been reported to dissociate on the surfaces of gold nanocrystals to form surface-bound thiolates and adsorbed hydrogen.^68^ Furthermore, previous work by De Roo et al. demonstrates that carboxylate and phosphonate ligands bound to metal oxide nanocrystal surfaces in nonpolar solvents can be charge-balanced by protons bound to adjacent surface oxygen moieties.^16,17^ We suspect that addition of oleic acid to CGO nanocrystals also produces oleate ligands along with surface-bound protons and that the thiolate proton is similarly bound to the oxide surface upon introduction of thiol. Such binding would maintain charge neutrality upon displacement of the neutral oleylamine ligand from CGO-OAm by 1-decanethiol and may enable proton-mediated X-type ligand exchange of thiolate for oleate on CGO-OA.^69,70^

We performed X-ray photoelectron spectroscopy (XPS) on the pre- and post-decanethiol exchanged samples (CGO-OAm sample after one ethanol wash and CGO-OAm-DT sample after one methanol wash, respectively) to gain insights into the surface chemical compositions and atomic valence states of the nanocrystals. The Co 2p XPS spectra can be deconvoluted into four peaks for both CGO-OAm (Figure 6A) and CGO-OAm-DT (Figure 6B). In the CGO-OAm sample, the peaks centered around 796.5 and 780.6 eV correspond to Co 2p_1/2_ and Co 2p_3/2_, respectively,^71^ and are consistent with the Co^2+^ oxidation state.^71^ Two satellite peaks around 802.2 and 784.7 eV are associated with the shakeup excitation of the high-spin Co^2+^ ions.^71^ Similar peaks are also present in CGO-OAm-DT, as seen in Figure 6B and Table 1. The absence of any significant changes in the binding energies of the spin–orbit doublet in Co 2p XPS spectra suggests that there is no change in the oxidation state of the cobalt ions after 1-decanethiol ligand exchange.

**Table 1:** Binding Energies of the Spin-Orbit Doublet in Co 2p XPS Spectra

Samples	Co 2p_1/2_ (sat)	Co 2p_1/2_	Co 2p_3/2_ (sat)	Co 2p_3/2_
CGO-OAm	802.2 eV	796.5 eV	784.7 eV	780.6 eV
CGO-OAm-DT	802.8 eV	796.5 eV	784.8 eV	780.5 eV

**Figure 6:**
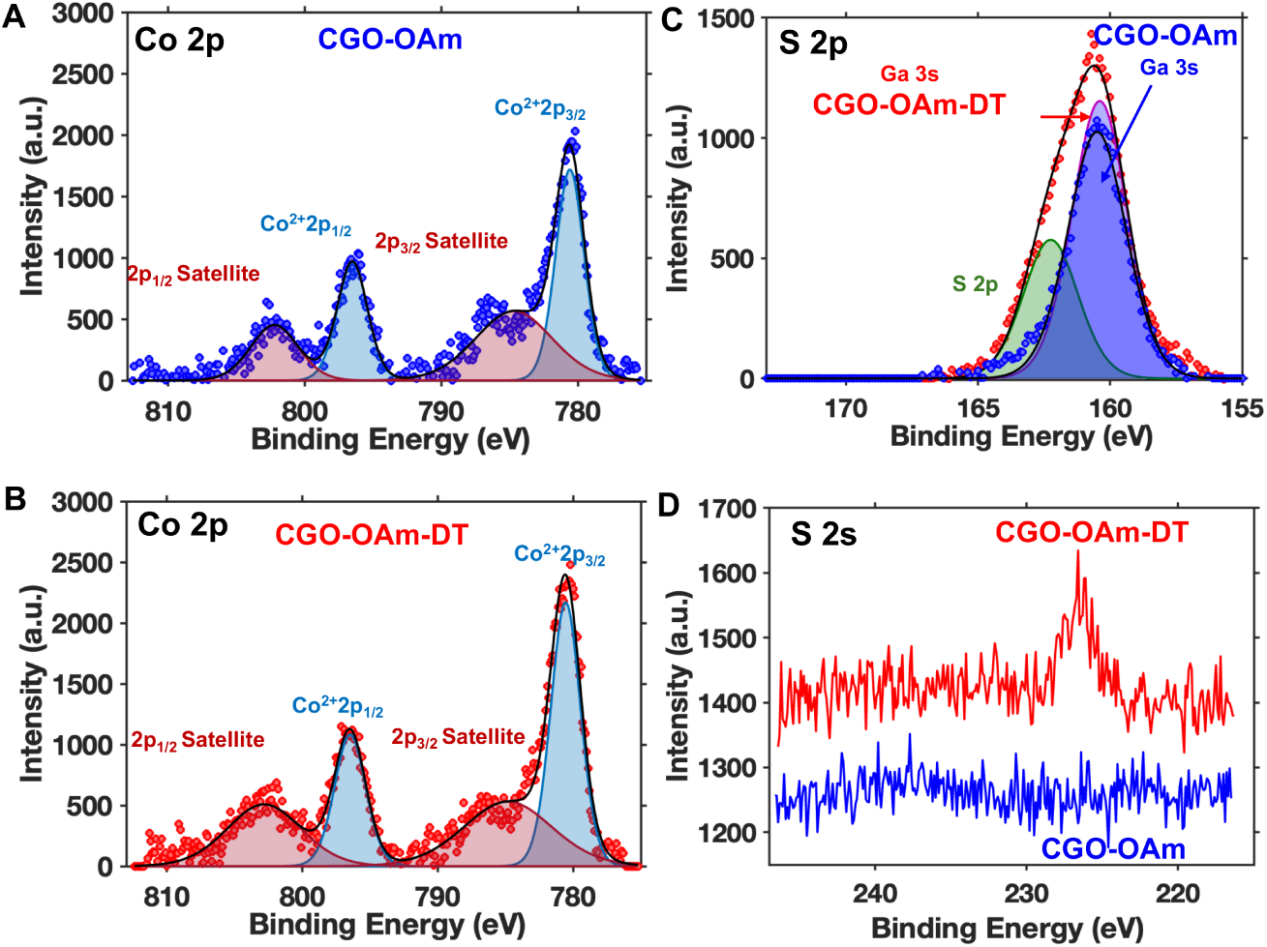
Co 2p XPS spectra of CGO-OAm (A, blue circles) and CGO-OAm-DT (B, red circles). (C) S 2p XPS spectra of CGO-OAm (blue circles) and CGO-OAm-DT (red circles). (D) S 2s XPS spectra of CGO-OAm (blue) and CGO-OAm-DT (red).

Figure 6C, D shows the XPS spectra of the S 2p and S 2s regions, respectively, of CGO nanocrystals before and after the 1-decanethiol ligand exchange. No sulfur peaks are observed in the CGO-OAm, however, the S 2p and S 2s spectra of CGO-OAm-DT contain evidence of the presence of sulfur. A peak corresponding to S 2p appears as a higher-energy shoulder of the Ga 3s peak of washed CGO-OAm-DT; the Ga 3s peak in CGO-OAm does not exhibit this peak. This shoulder is centered at a binding energy of 162.2 eV in CGO-OAm-DT, which is consistent with the presence of chemisorbed thiolates after the decanethiol ligand exchange.^37,72,73^ The sulfur 2s region contains a peak centered around 225 eV in CGO-OAm-DT, which is absent in CGO-OAm. There are no significant differences between the Ga 2p, Ga 3d, O 1s, and C 1s regions of the pre- and post-DT ligand-exchanged samples (see Figure S11), indicating that the valence states of these elements remain the same even after the addition of 1-decanethiol. Overall, these data are consistent with the appearance of surface-bound sulfur-containing species upon the addition of 1-decanethiol to CGO-OAm nanocrystals. Importantly, these species are not removed upon washing with methanol (see Figure S10B,C).

In order to further evaluate changes in the oxidation state and the coordination environment of cobalt in CGO nanocrystals before and after addition of 1-decanethiol, we explored the Co K-edge absorption of our washed pre- and post-ligand-exchanged samples (CGO-OAm and CGO-OAm-DT washed) via X-ray near-edge absorption spectroscopy (XANES) as observed in Figure 7. The Co K-edge fingerprint is quite sensitive to changes in local coordination and oxidation states, allowing detailed description of the electronic structure of our samples.

**Figure 7:**
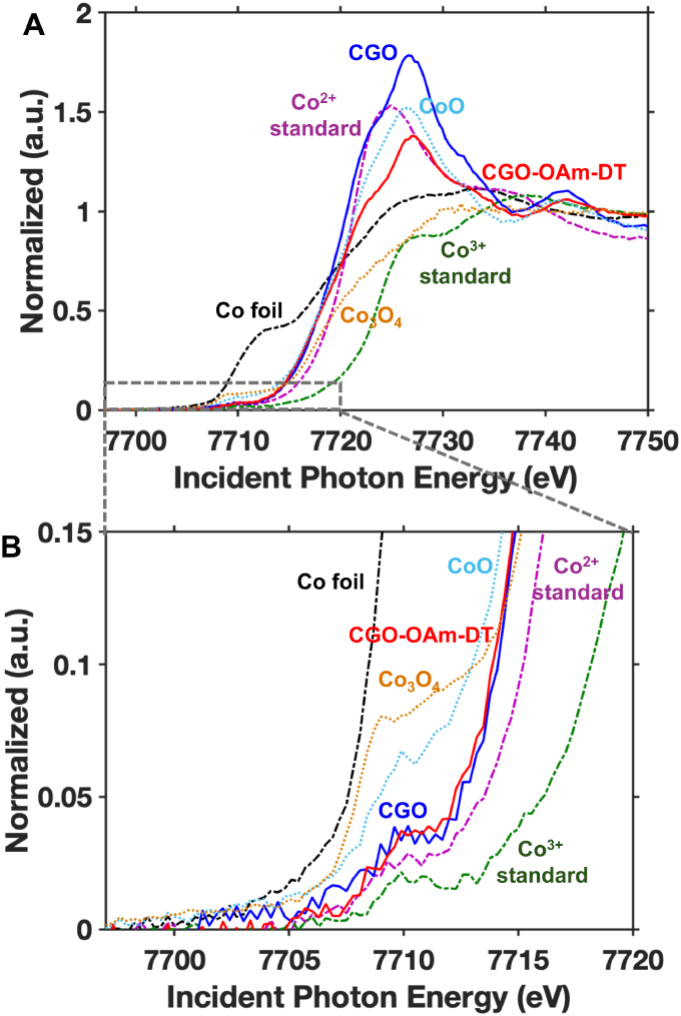
(A) Pre- and post-edge normalized Co K-edge XANES of CGO nanocrystals before and after DT ligand exchange, reference standards Co foil, Co­(acac)_2_, Co­(acac)_3_, CoO (rock salt) and Co_3_O_4_ spinel. (B) Zoomed in XANES data highlighting the pre-edge region.

Figure 7A depicts the XANES spectra obtained for the Co K-edge for seven samples that include Co metal foil, two precursor standards, CoO, Co_3_O_4_, and the pre- and post-ligand-exchanged samples. As a first observation, it is clear that none of the samples present Co^0^ character due to the presence of pre-edge features. To evaluate the global oxidation state of our samples, the Co^2+^ precursor (Co­(acac)_2_) and CoO were used as standards for Co^2+^. These standards exhibit a main peak at 7,724.9 eV and 7,726.4 eV, respectively. Whereas the Co^3+^ standard (Co­(acac)_3_) shows two peaks at 7,727.9 eV and 7,738.7 eV, and the mixed-valence standard Co_3_O_4_ (Co^2+/3+^) shows a broad peak at 7,730.9 eV. These peaks correspond to transitions from the 1s to the 4p states for these samples.^74^ The pre- and post-ligand exchanged CGO samples each show an absorbance peak at 7,726.9 eV. In previous reports of cobalt gallate nanoparticles, an absorption peak at this energy position was assigned to Co^2+^ with an additional contribution of some small amounts of Co^3+^ coming from the partially inverted spinel structure CoGa_2_O_4_ can stabilize.^31^ Indeed, this energy position has also been assigned to Co^2+^ in a wurtzite crystal structure in Co-doped ZnO,^75^ suggesting that the global oxidation state of Co in our material is 2+. This observation is confirmed when comparing our samples to the fingerprint of CoO. The decrease in normalized intensity observed for the CGO-OAm-DT sample compared to CGO-OAm could be related to the more electron-donating nature of the sulfur in 1-decanethiol compared to the nitrogen in oleylamine.^76^ The decrease in intensity can be related to empty Co 4p states being partially occupied correlating to the intrinsic relationship the bound ligand has to the electronic nature of Co.^74,77,78^

The weak pre-edge peak at around 7,710.2 eV for the cobalt K-edge corresponds to dipole-forbidden transitions from the 1s to 3d orbitals that become allowed due to hybridization of 4p-states from neighboring cobalt atoms and can reflect changes in the local symmetry.^74,79^ The pre-edge of the Co­(acac)_2_ standard predominantly begins at about 7707.5 eV, before that of the Co­(acac)_3_ standard at about 7,708.5 eV even though both start deviating from 0 around 7700 eV. The normalized intensity changes between the Co^2+^ and Co^3+^ standards is related to the differing coordination environments: Co^2+^ has a tetrahedral coordination environment, whereas the Co^3+^ standard has an octahedral coordination environment. Normalized intensity differs among these symmetries due to changes in *p-d* orbital hybridization, which is more apparent in lower symmetries. In comparison, we observe that the intensity of the pre-edge varies between Co_3_O_4_ (Co^2+/3+^) and CoO (Co^2+^), further demonstrating the effects of changes in coordination and oxidation state on the pre-edge features. Thus, for these states to be allowed, the excited electron from the 1s shell must transition to a state with p-character (dipole selection rules), which is more accessible in tetrahedral complexes. The presence of the pre-edge in both the pre- and post-decanethiol ligand exchanged CGO samples, but with rather low intensity, confirms the presence of Co^2+^ within both tetrahedral and some octahedral coordination.^31,75,79^ Indeed, this observation is representative of the partially inverted spinel nature of CoGa_2_O_4_.^31,80–82^ Previous reports have connected the extent of tetrahedral and octahedral distortions at the cobalt site in CGO nanocrystals to changes in color;^31^ however, our work demonstrates that control over the color of CGO nanocrystals can be achieved through changes in ligation at the nanocrystal surface without major changes in the global coordination and oxidation state of cobalt.

### Optical Changes in CGO Nanocrystals Can Be Reversed Upon Replacing Thiol Ligands with Oleic Acid

To further confirm that the optical changes we observed arise from replacement of oleylamine or oleic acid ligands with thiol ligands, we attempted “reverse” ligand exchange reactions to displace the surface-bound thiols. The data from Figure 4 indicate that oleic acid is harder for 1-decanethiol to displace than oleylamine, which implies that oleic acid may be able to displace the thiol. Upon addition of oleic acid to thiol-exchanged nanocrystals (CGO-OAm-DT) in toluene at room temperature, no color changes are observed (see Figure S12). However, upon heating CGO-OAm-DT nanocrystals to reflux in toluene (∼115 °C) in the presence of oleic acid and subsequent washing with methanol, we observe recovery of the original blue color associated with as-synthesized CGO and disappearance of the peak centered at 2.4 eV after 24 h (Figure 8A). ^1^H NMR spectra after the reversibility experiment confirm that the ligand exchange with oleic acid was successful—only broadened peaks matching oleic acid resonances are observed (Figure 8C). Both the intensity of the 2.4 eV feature and the dark red color are retained upon heating CGO-OAm-DT to reflux in toluene without addition of oleic acid (Figure 8B), consistent with our hypothesis that the return to the original blue-green color results from displacement of thiol ligands with oleic acid. Heating CGO-OAm-DT in toluene with or without addition of oleic acid did not significantly impact the crystal structure or morphology of the nanocrystals (see Figure S13). We also attempted to displace decanethiol from CGO-OAm-DT by adding oleylamine at 115 °C. This reaction also successfully removed the feature at 2.4 eV, but the resulting nanocrystals were not very colloidally stable (see Figure S14). Finally, we found that heating CGO-OAm-DT nanocrystals to higher temperatures (∼130 °C) in octadecene resulted in the recovery of the blue-green color after only 10 min regardless of whether oleic acid or oleylamine is added (see Figure S15). These data indicate that the surface-bound thiol ligands are not very stable at elevated temperature; however colloidal dispersions of CGO-OAm-DT in tetrachloroethylene show no visible precipitation or changes in optical properties after storage for three months under ambient conditions (Figure S16). Upon refunctionalizing these reversed nanocrystals (reversed CGO-OAm-DT in toluene) with 1-decanethiol, optical features identical to what was observed upon the original 1-decanethiol exchange (i.e., in CGO-OAm-DT) were observed (see Figure S17). Overall, these experiments demonstrate that the optical changes observed upon 1-decanethiol addition are reversible upon displacing thiols from the surface.

**Figure 8:**
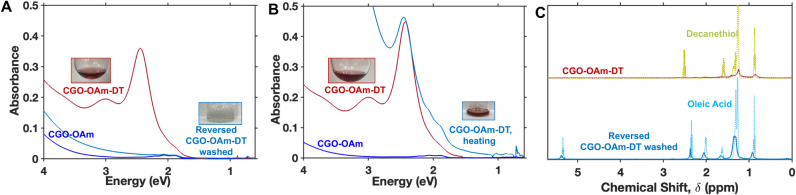
(A) UV-vis absorption spectra of CGO-OAm (dark blue), CGO-OAm-DT (dark red), and the product of the “reverse” ligand exchange reaction between CGO-OAm-DT and oleic acid (light blue). (B) UV-vis absorption spectra of CGO-OAm (dark blue), CGO-OAm-DT (dark red), and the product obtained from heating CGO-OAm-DT in toluene in the absence of oleic acid (light blue). (C) ^1^H NMR spectra of CGO-OAm-DT (dark red) and the product of the “reverse” ligand exchange reaction between CGO-OAm-DT and oleic acid (solid light blue) along with the spectra of free 1-decanethiol (dashed yellow-green) and free oleic acid (dashed light blue).

### Origin of Optical Changes Observed Upon Addition of Thiol

Based on the results from ^1^H NMR, XPS, and XANES measurements and analysis, and the observed reversibility of the optical changes, we conclude that surface-bound cobalt-thiolate species are responsible for the optical changes we observe upon addition of thiol ligands to CGO nanocrystals. XPS and XANES measurements both indicate that cobalt is present primarily in the 2+ oxidation state, but we cannot rule out the presence of a small percentage of Co^3+^. However, addition of decanethiol to Co­(acac)_2_ or Co­(acac)_3_ results in the appearance of an intense absorption feature centered at 2.4 eV in both cases albeit the absorption is much more intense for Co­(acac)_2_ compared to Co­(acac)_3_ (Figure S18A); this feature is similar to the absorption feature we observe upon addition of 1-decanethiol to CGO nanocrystals. This control experiment suggests that the optical response observed upon thiol binding does not discriminate between Co^2+^ and Co^3+^ species. Similarly, the crystal field transitions observed at ∼2.0 and ∼0.8 eV in the optical spectra of CGO nanocrystals (Figure 1D) indicate the presence of Co^2+^ within tetrahedral (A) sites while XANES analysis (Figure 7) indicates that Co^2+^ also occupies octahedral (B) sites. Indeed, bulk CoGa_2_O_4_ has been reported to have an inversion parameter of ∼0.6, corresponding to ∼60% of the cobalt ions occupying octahedral sites.^80–82^ Nevertheless, we strongly suspect that the optical response of CGO nanocrystals to the binding of thiol ligands does not depend on the distribution of cobalt ions among octahedral and tetrahedral sites for two reasons: (i) The coordination environment of surface cobalt ions is different from bulk cobalt ions and depends not only on whether cobalt occupies an A-site or a B-site but also on the surface facet and the number and type of bound surface ligands. It is this surface coordination environment, rather than the bulk coordination, that is relevant to the interaction between surface cobalt ions and thiol ligands. (ii) Both tetrahedral and octahedral molecular Co^2+^ complexes produce a similar optical feature in response to the addition of 1-decanethiol (see Figure S18).

To further corroborate the link between the appearance of an intense absorption feature that appears at 2.4 eV in CGO nanocrystals upon addition of thiol to the formation of cobalt-sulfur bonds at the nanocrystal surface, we turn to previous reports of the optical properties of both molecular and surface-bound cobalt-thiolate species. Studies of molecular cobalt­(II) thiolate complexes report the presence of both d-d and charge transfer transitions in the visible region of the spectrum. A series of homoleptic benzenethiolate cobalt complexes reported by Dance in 1979 (namely [(μ-SPh)_6_(CoSPh)_4_]^2–^, [(μ-SPh)_6_(CoSPh)_2_(CoCl)_2_]^2–^ , and [Co­(SPh)_4_]^2–^) contain both low energy and high energy charge transfer transitions that roughly correspond to the two primary features we observe in CGO–DT at 2.4 and 3 eV.^83^ However, unlike our observations, in these molecular systems the intensity of the higher-energy features dominate, producing complexes that are deep green or brown in color. A series of analogous ethylenethiol complexes reported by Holm in 1984 exhibits similar spectral properties.^84^ Recently, Cook et al. reported the synthesis of a tetrahedral cobalt thiolate cluster [Co_10_(SCH_2_CH_2_Ph)_16_Cl_4_].^40^ Under inert conditions, the absorption spectrum of this complex resembles those reported by Dance and Holm. However, upon exposure to oxygen, the complex changes color from brown to coral and develops an absorption spectrum that closely resembles that of our CGO–DT sample (Figure 9). The authors posit that air exposure produces a mixture of Co_*x*_O_*y*_(SCH_2_CH_2_Ph) clusters. Bhattacharyya et al. report a cobalt­(II) complex of a pentadentate N_4_S ligand that contains an absorption feature centered at 450 nm (2.76 eV) that the authors assign to a transition of mixed d-d and LMCT character; specifically a d-d transition that borrows intensity from the tail of a nearby S → Co^II^ LMCT band centered at 371 nm (3.35 eV).^85^

**Figure 9:**
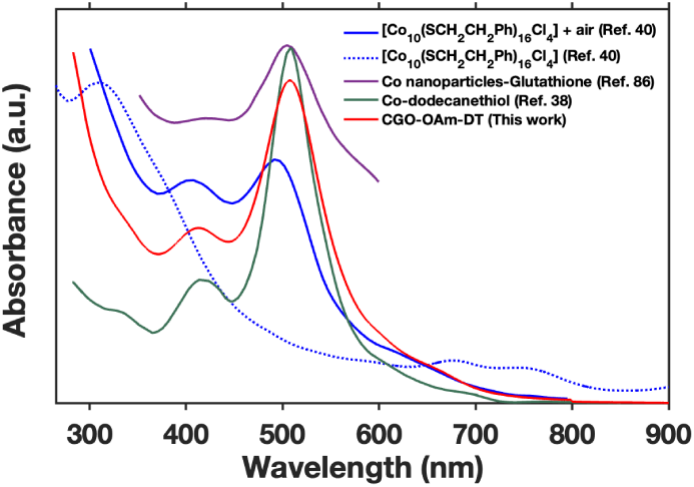
UV-vis spectra of various thiolated cobalt species from reported in the literature compared with the spectrum of CGO-OAm-DT.

In addition to molecular cobalt-thiolate complexes, cobalt-thiolate species with similar optical spectra to CGO-DT have been obtained upon treating metallic cobalt nanocrystals or microcrystals with thiol ligands. Co nanocrystals capped with mercaptoethanol, mercaptopropanoic acid, or glutathione all exhibit intense absorption peaks ranging from 580 to 520 nm, consistent with cobalt-thiolate species (Figure 9).^39,86^ Addition of alkanethiols to micron-sized crystals of metallic cobalt produced a composite material comprising nanometer-sized cobalt clusters bound to thiol ligands and embedded in a polymeric cobalt-thiolate matrix.^38^ The resulting composite has an absorption spectrum similar to CGO-DT (Figure 9).

From these previous reports, it is clear that the optical properties of our CGO-DT samples more closely resemble those of other nanoparticle-bound cobalt-thiolate species than the molecular cobalt complexes. Although the exact nature of the optical transitions in the previously reported nanoscale systems has not been definitively assigned, given the significantly larger intensity of the feature centered at 2.4 eV compared to the ligand field transitions at 1.8–2.3 eV and 0.8–1 eV, we propose that the 2.4 eV transition has primarily charge transfer character, and perhaps involves some mixing of d-d character with LMCT character as proposed by Bhattacharyya et al.^85^

Finally, we consider the response of NiGa_2_O_4_ nanocrystals to addition of 1-decanethiol. Analogous to CGO-DT, the optical features observed in NGO-DT, particularly the intense peak centered at 3.7 eV in the UV, are also present in the optical spectra of polynuclear nickel­(II) thiolate complexes and appear upon addition of alkanethiols to metallic Ni nanocrystals.^37,87–89^ We therefore strongly suspect that addition of 1-decanethiol to NiGa_2_O_4_ nanocrystals results in the formation of nickel thiolate species. However, unlike CoGa_2_O_4_, NiGa_2_O_4_ does not exhibit any response to addition of MSA and MPA. Furthermore, unlike CoFe_2_O_4_, NiFe_2_O_4_ does not exhibit any optical changes upon addition of 1-decanethiol. These inconsistencies suggest an additional level of complexity in the factors that govern the ability of thiol ligands to bind to nickel-containing spinel nanocrystals, the unraveling of which is beyond the scope of this study.

## Conclusions

This work demonstrates that surface-bound species can dominate the absorption spectra of colloidal transition metal oxide nanocrystals. The intense optical features observed in the cobalt-containing nanocrystals after thiol functionalization are due to the formation of cobalt thiolate linkages on the surface of the nanocrystal, and correspond to surface-localized excitations. Creation of surface-localized states on these nanocrystals with strong optical absorptions could potentially lead to enhanced photocatalytic reactivity as the excited charge carriers are close to the surface and hence have less of a chance to recombine before they interact with reactants. We also demonstrated that the observed optical change could be used as a tool to track ligand exchange reactions and assess the relative binding affinity of thiol, amine, and carboxylate ligands to the nanocrystal surface.

## Supplementary Material



## Notes

Structural characterization (powder XRD, TEM) of MGa_2_O_4_, MFe_2_O_4_, and CGO-OAm-DT nanocrystals, elemental analysis of CGO-OAm and CGO-OAm-DT nanocrystals, determination of the per-Co extinction coefficient of CGO, ^1^H NMR of CGO-OAm and CGO-OA, Raman spectra of CGO and CGO-OAm-DT, additional ligand exchange control experiments, additional XPS characterization of CGO-OAm and CGO-OAm-DT, additional reverse ligand experiments, and readdition of thiols (PDF)
