# Supplementary material for: Ligand-Dependent Optical Properties of Colloidal Ternary Spinel Oxide Nanocrystals Containing Transition Metals
Source: Inorg Chem. 2025 Jul 16;64(29):15152–64. doi: 10.1021/acs.inorgchem.5c02179 (PMC12308797; doi:10.1021/acs.inorgchem.5c02179)
Supplement: Supplementary file 1 [file ic5c02179_si_001.pdf]

Supporting Information for

Ligand-Dependent Optical Properties of Colloidal Ternary Spinel Oxide Nanocrystals containing Transition Metals

Revathy Rajan[1], Jordan C. Scalia[2], Luis R. De Jesús Báez[2], Kathryn E. Knowles*[1]

[1]*Department of Chemistry, University of Rochester, Rochester, New York 14627, United States*

[2]*Department of Chemistry, University at Buffalo – The State University of New York, Buffalo, New York, 14260-3000, United States*

*corresponding author. E-mail: kknowles@ur.rochester.edu

Determination of Lattice Constant of CGO

The lattice parameter (a) of CGO nanocrystals was determined from the positions of diffraction peaks in the powder X-ray pattern using equations S1 and S2.

$$d_{hkl} = \frac{\lambda}{2sin\theta} \tag{S1}$$

$$a = d_{hkl}\sqrt{h^2 + k^2 + l^2} \tag{S2}$$

In these equations, d_{hkl} is the d-spacing, λ is the wavelength of the X-ray source (here we use Cu Kα, λ = 1.54158 Å), θ is the Bragg angle, and *hkl* are the Miller indices associated with each diffraction peak. We calculated values of lattice parameters from the five most intense peaks in the XRD pattern and report their average in the main text. Table S1 contains the complete set of data used in these calculations.

Table S1. Data extracted from Figure 1B and used to calculate the lattice parameter of CGO.

**λ (Å)**	**Miller indices**			**Bragg's angle (°)**		**d-spacing (Å)**	$\sqrt{(h^2 + k^2 + l^2)}$	**Lattice constant (Å)**	**Average a (Å)**
	h	k	l	2θ	θ				
1.54158	2	2	0	30.56	15.28	2.9245	2.8284	8.273	8.272
1.54158	3	1	1	36.02	18.01	2.4930	3.3166	8.268	
1.54158	4	0	0	43.74	21.87	2.0692	4	8.277	
1.54158	5	1	1	57.92	28.96	1.5920	5.1962	8.272	
1.54158	4	4	0	63.65	31.83	1.4617	5.6569	8.269	

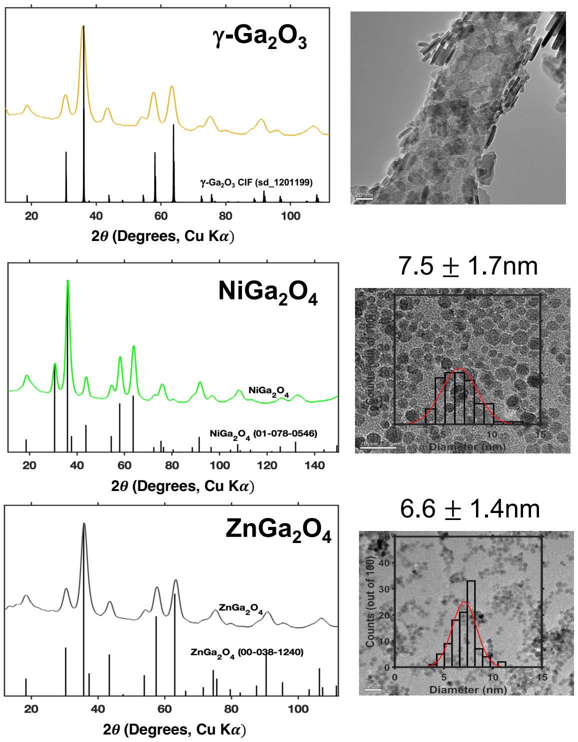

Figure S1. Powder XRD patterns and TEM images of as-synthesized γ-Ga_2O_3, $NiGa_2O_4$ and $ZnGa_2O_4$ nanocrystals. The black vertical lines in the XRD patterns represent standard patterns for these materials extracted from the JCPDS database except for γ-Ga_2O_3 (produced from Vesta using the CIF file with dataset ID sd_1201199[1]). All three samples have patterns that match those of the standards, indicating that they are phase-pure spinels. The γ-Ga_2O_3 nanocrystals exhibit platelet-like morphology whereas the $NiGa_2O_4$ and $ZnGa_2O_4$ nanocrystals are quasi-spherical with diameters of 7.5 ± 1.7 nm and 6.6 ± 1.4 nm, respectively. These sizes represent the average and standard deviation of 100 nanocrystals. The scale bars in the TEM images are 20 nm.

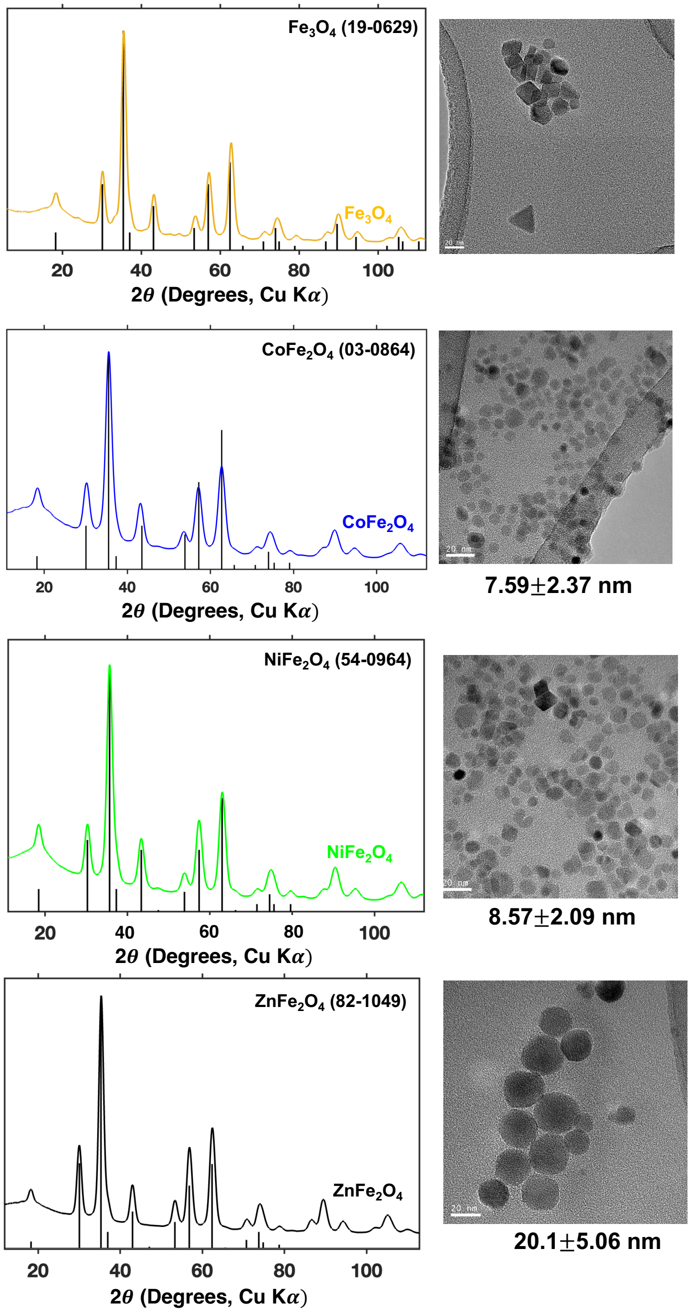

Figure S2. Powder XRD patterns and TEM images of as-synthesized Fe_3O_4, $NiFe_2O_4$ and $ZnFe_2O_4$ nanocrystals. The black vertical lines in the XRD patterns represent standard patterns for these materials extracted from the JCPDS database. All samples have patterns that match those of the standards, indicating that they are phase-pure spinels. The scale bars on the TEM images each correspond to 20 nm.

Elemental Analysis and Determining the per-Co extinction coefficient of CGO

Atomic absorption measurements

An absorption spectrum of a 5mg/mL or 3 mg/mL colloidal solution of $CoGa_2O_4$ nanocrystals in tetrachloroethylene (TCE) was collected. Either 50 µL or 100 µL of this solution was dried with a flow of nitrogen to remove the TCE, and the resulting solid was digested with 0.5 mL concentrated nitric acid and then 15 mL of Nanopure water was added. Known standards with cobalt concentrations ranging from 0 to 4.5 ppm were prepared by diluting a standard cobalt solution (100 ± 0.6 ppm from High-Purity Standards) with a 0.2% nitric acid solution in Nanopure water. Atomic absorption (AA) measurements were done on a Shimadzu atomic absorption spectrophotometer (AA-7000 series) using a hollow cathode Co lamp (L2433-27NU). First, the standards were used to obtain the calibration curve shown in Figure S3A (green stars), which was fitted to a linear curve (black line). Subsequently, the $CoGa_2O_4$ nanocrystal samples from four different synthetic batches (two samples per batch) were measured to obtain the concentration of Co.

ICP-MS measurements

ICP-MS was carried out on a Perkin Elmer 2000C ICP-MS in KED mode at 4 mL/min helium flow. The power used was 1600 watts, the nebulizer flow was 0.95mL/min, and the auxiliary argon flow was 1.2 mL/min. The plasma argon flow was 15 L/min. Co-59, Ga-69, and S-34 isotopes were analyzed. Calibration standards for Co, Ga, and S were prepared by diluting standard solutions as described for the AA measurements (Ga standard: 1000 ± 4 ppm from High-Purity Standards, S standard: 1000 ppm from SPEX CertiPrep). CGO samples were prepared as described for the AA measurements. The sample of CGO-OAm-DT for ICP-MS measurement was prepared by taking 500 µL of the 5mg/ml CGO-OAm-DT washed solution, drying with a flow of nitrogen to remove the TCE, and digesting with 0.5 mL aqua regia. The digestion solution was diluted with 15 mL of Nanopure water.

Knowing the concentrations of Co obtained from both AA and ICP data and the corresponding electronic absorption spectra, the per Co molar extinction coefficient was determined from the Beer-Lambert Law. Table S2 lists the average values of the molar extinction coefficients at each

of the three peaks in the higher-energy d-d band (1.9-2.2 eV). The uncertainties are calculated as the standard deviation of the combined measurements across all synthetic batches. The value of this extinction coefficient shows negligible batch-to-batch variability. Figure S3B shows the extinction spectra plotted with standard deviation at each wavelength obtained from a total of 10 measurements (8 AA and 2 ICP).

Table S2. Average extinction coefficient values calculated from different synthesis batches.

Energy (eV)	Average Epsilon values ($M^{-1}cm^{-1}$)
2.20	31 ± 2
2.06	45 ± 4
1.89	41 ± 4

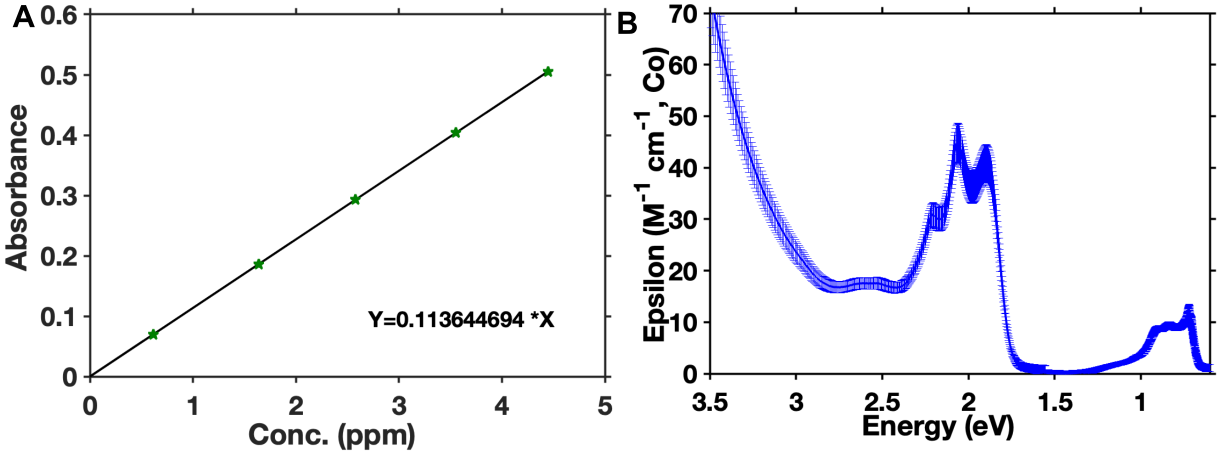

Figure S3. A) Calibration curve showing the absorbance dependence on the concentration of standard solutions of cobalt (green stars), obtained from atomic absorption spectroscopy. The black line shows the best-fit line. **B)** Extinction spectra with standard deviation obtained from multiple trials across four different synthetic batches of CGO.

Determination of the surface density of thiol ligands in CGO-OAm-DT

ICP-MS analysis of a washed sample of CGO-OAm-DT revealed a S:Co:Ga ratio of 0.07:0.7:2. Due to the Co-deficient stoichiometry of CGO, we use the S:Ga ratio of 0.035:1 to estimate the surface density of thiol ligands as follows:

1. Compute the number of Ga atoms per nanocrystal using the lattice parameter a = 8.272 Å, nanocrystal radius (assuming a spherical geometry), and assuming 16 Ga per unit cell for the spinel

structure. This calculation gives a range of 3880-18600 Ga per NC for radii ranging from 3.2-5.4 nm. This range of radii corresponds to the average radius (4.3 nm, 8400 Ga/NC) $\pm \sigma = 1.1$ nm.

2. Multiply by the measured S:Ga ratio of 0.035 to determine the number of S atoms per nanocrystal.

3. Divide the number of S atoms by the surface area of a sphere of radius 3.2-5.4 nm.

These calculations give an estimated surface density of 1.4 ± 0.4 nm^{-2}.

High resolution TEM

We were able to resolve the (111), (311), and (220) planes and their corresponding d-spacings in high-resolution TEM images of CGO. The d-spacings determined from high-resolution TEM are in good agreement with the d-spacings obtained from powder XRD. There is only a small difference of less than ~ 0.03 Å between the two techniques and hence we can confirm the phase purity of our sample.

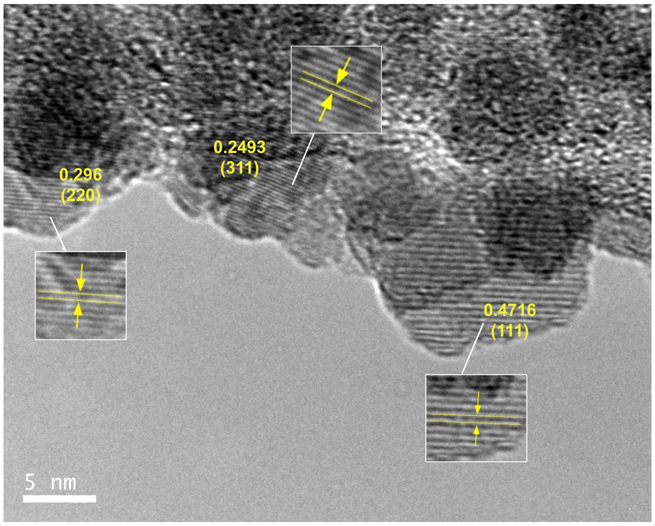

Figure S4. High-resolution TEM images of $CoGa_2O_4$ nanocrystals with d-spacings for the (111), (311), and (220) planes are resolved. Scale bars correspond to 5 nm.

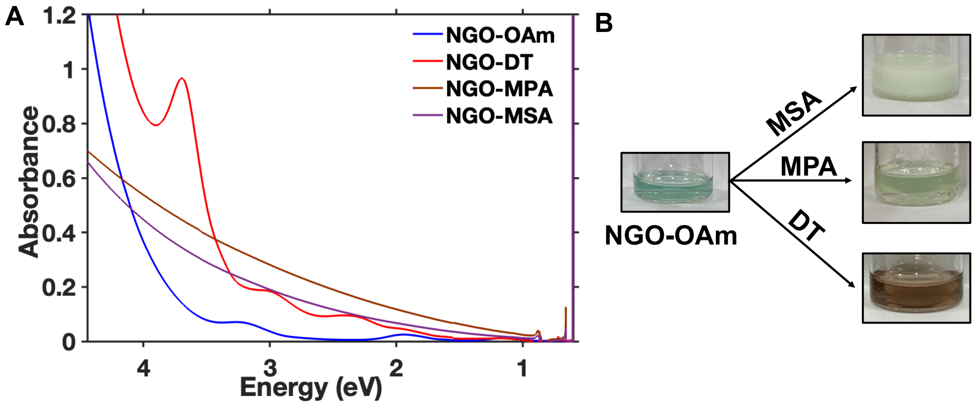

Figure S5. A) UV-Vis Spectra of $NiGa_2O_4$ nanocrystals (NGO-OAm) before and after the addition of thiol ligands (105 µL of DT, 76 mg of MSA, or 44 µL of MPA was added to 2 mL of a 10 mg/mL suspension of NGO-OAm). Blue: As-synthesized $NiGa_2O_4$ capped with oleylamine in hexane (NGO-OAm), Purple: $NiGa_2O_4$ after addition of mercaptosuccinic acid and re-dispersion in water (NGO-MSA), Maroon: $NiGa_2O_4$ after addition of mercaptopropionic acid and re-dispersion in water (NGO-MPA), Red: $NiGa_2O_4$ after addition of 1-decanethiol in hexane (NGO-DT). **B)** Photographs of $NiGa_2O_4$ nanocrystal suspensions before (left) and after addition of thiol ligands.

Ligand exchange reactions with alcoholic counterparts: Succinic acid (SA), Malic Acid (MA) and Propionic Acid (PA):

To a 2 mL colloidal solution of 10 mg/mL $CoGa_2O_4$ (CGO), $NiGa_2O_4$ (NGO), or $ZnGa_2O_4$ (ZGO) in hexane, an amount of ligand corresponding to approximately 1:50 molar ratio of Co to ligand for CGO was added to the mixture (i.e 0.29 g SA, 0.34 g MA, 190 μL PA). No significant changes in color were observed, however, the dispersions became more turbid, indicating a change in dispersibility as a result of ligand exchange at the surface.

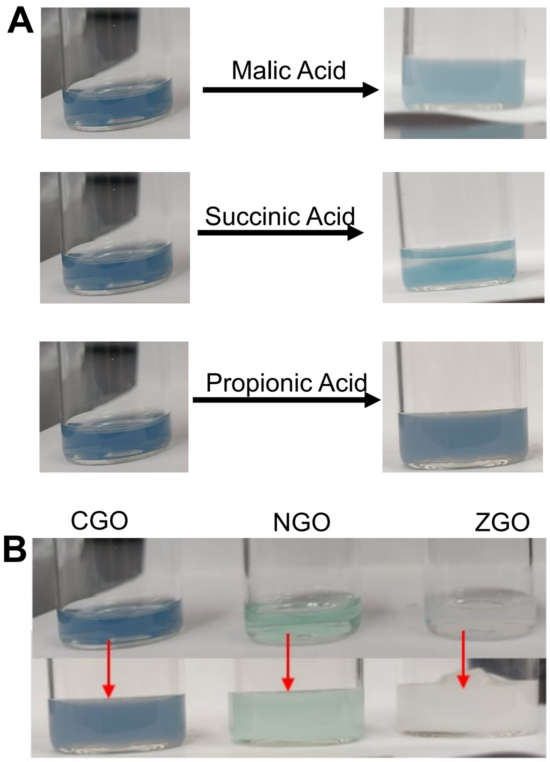

Figure S6. A) Photographs of $CoGa_2O_4$ before and after addition of malic acid, succinic acid and propionic acid (PA) ligand exchange **B)** Photographs of $CoGa_2O_4$, $NiGa_2O_4$ and $ZnGa_2O_4$ before and after propionic acid ligand exchange.

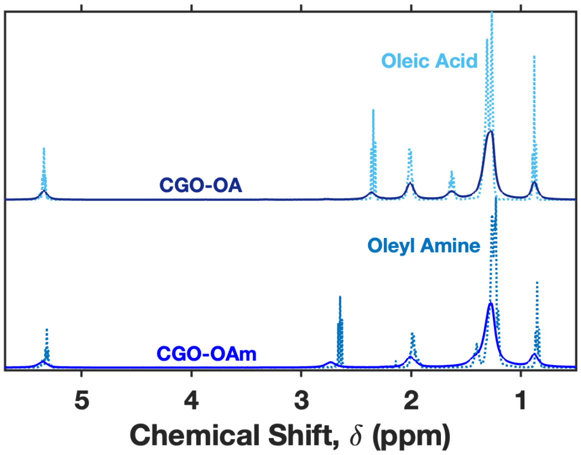

Figure S7. ^{1}H NMR spectra of oleate- and oleylamine-capped $CoGa_2O_4$ (CGO-OA and CGO-OAm respectively) plotted with ^{1}H NMR spectra of the corresponding free ligands (oleic acid and oleylamine).

Raman spectroscopy

The CGO-OAm-DT washed and CGO sample in hexane was drop-cast onto a roughened gold-plated glass. Stokes Raman spectra were collected using a back-scattering set-up with a 532-nm CW diode laser and Princeton Instruments monochromators equipped with a CCD detector. Laser power was kept at < 30 mW to limit the possibility of sample heating. The Raman shift axis of the CCD for sample data was corrected with a cyclohexane spectrum.

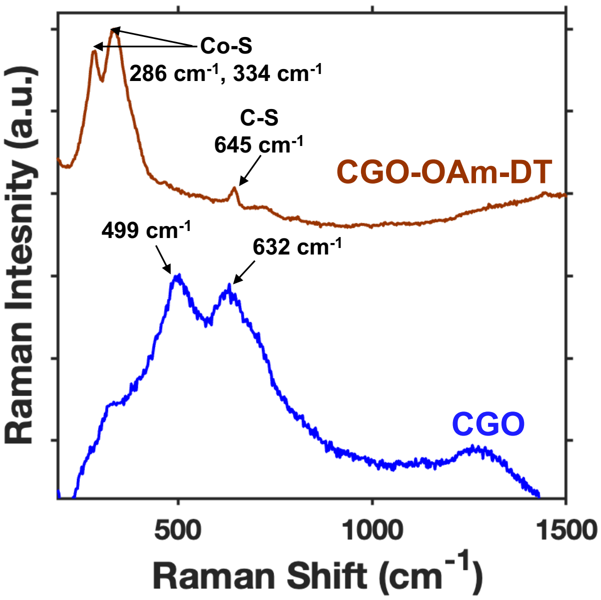

Figure S8. Stokes Raman spectrum of CGO-OAm-DT and CGO nanocrystals dropcasted onto a roughened gold substrate.

Figure S8 shows a Stokes Raman spectrum of a washed CGO-OAm-DT sample that contains peaks at 286 cm^{-1} and 334 cm^{-1}, which correspond to the symmetric Co-S stretch and the asymmetric Co-S stretch, respectively.[2-5] The peak at 645 cm^{-1} can be assigned to the C-S stretch.[3] These peaks are not present in the Raman spectrum of the as-synthesized CGO sample, which contains two primary peaks at 499 cm^{-1} and 632 cm^{-1} corresponding to phonon modes within the $CoGa_2O_4$ lattice.[6] The Raman signals corresponding to $CoGa_2O_4$ modes observed in the CGO sample were much weaker than those assigned to Co-S modes observed in the CGO-OAm-DT sample, thus we suspect that in the CGO-OAm-DT spectrum, the $CoGa_2O_4$ modes are masked by the Co-S and C-S features.

Thioether control experiment

To 10 mL of a 1 mg/mL solution of CGO-OAm in tetrachloroethylene, 50 µL of dibutyl sulfide (1:10 ratio of Co: dibutyl sulfide) was added. No significant changes to the absorption spectrum were observed upon thioether addition.

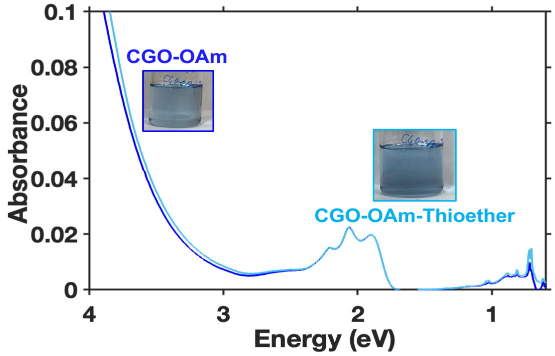

Figure S9. UV-Vis spectra of CGO-OAm in tetrachloroethylene before (dark blue) and after (light blue) addition of dibutyl sulfide.

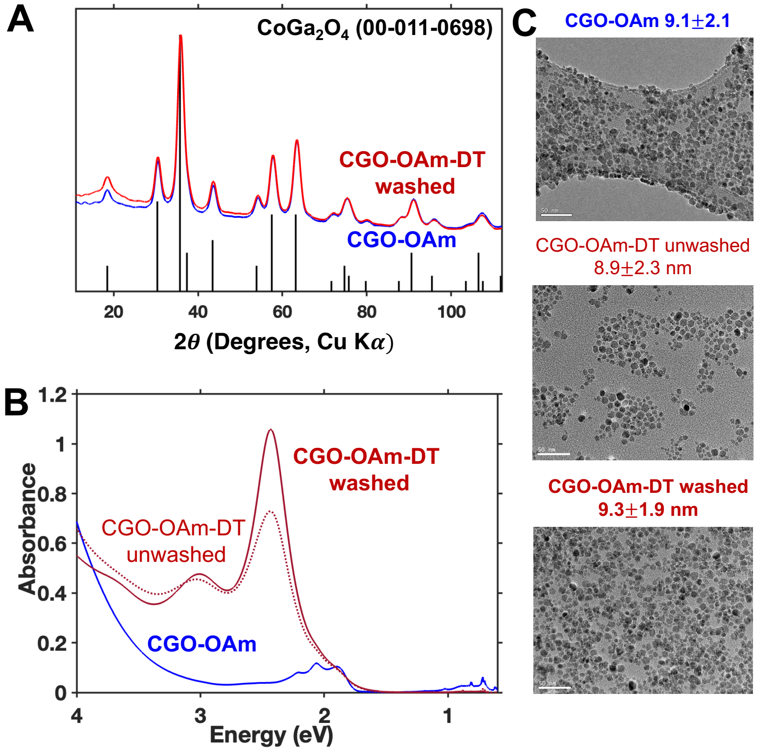

Figure S10: A) Powder X-ray diffraction patterns of CGO nanocrystals before (CGO-OAm, blue) and after (CGO-OAm-DT, red) decanethiol ligand exchange. **B)** UV-Vis spectra of CGO-OAm-DT before and after washing the ligand-exchanged sample with methanol. **C)** TEM images of

$CoGa_2O_4$ before and after the addition of decanethiol and washed with methanol. The scale bar for the CGO-OAm, CGO-OAm-DTunwashed and CGO-OAm-DT washed are 50 nm.

The powder XRD pattern of CGO-OAm-DT looks exactly like that of CGO-OAm, suggesting that the crystal structure remains the same even after the addition of DT and that the changes seen in the absorption spectra are due to changes in surface chemistry rather than changes in the structure of the nanocrystal core (Figure S10A). The optical features at 2.4 eV and 3 eV are preserved even after washing the DT ligand-exchanged samples with methanol (Figure S10B). Figure S10C shows TEM images of CGO before and after adding decanethiol. The CGO-OAm-DT washed sample is the decanethiol ligand exchanged sample, which is washed with methanol to remove free ligands present after the exchange (Figure 5 from the main text). Nanoparticles are present before and after the ligand exchange and after washing. The sizes of the pre and post-ligand exchanged samples were similar within the standard deviation limits (9.1 ± 2.1 nm before ligand exchange and 9.3 ± 1.9 nm after decanethiol ligand exchange after washing with methanol).

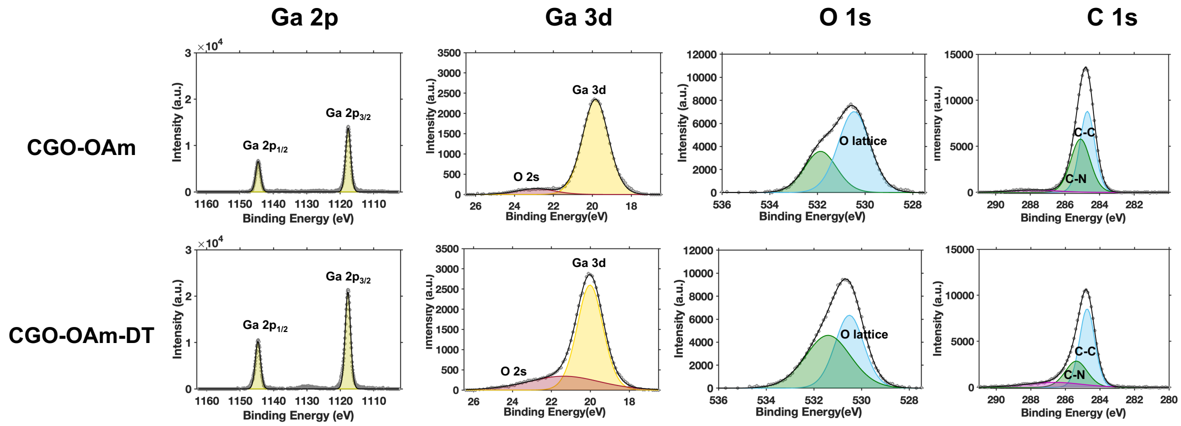

Figure S11. XPS spectra of the Ga 2p, Ga 3d, O 1s and C 1s regions measured for samples of CGO nanocrystals before (CGO-OAm, top row) and after (CGO-OAm-DT, bottom row) decanethiol ligand exchange (washed). These regions show little change upon addition of decanethiol. We note that the spin-orbit splitting of Ga 3d orbitals is only ~0.5 eV, which is smaller than the peak linewidths and therefore makes it difficult to unambiguously deconvolute the $3d_{5/2}$ and $3d_{7/2}$ peaks.[7,8]

Reversibility of Decanethiol Ligand Exchange Correlates with Reversibility of Optical Changes

Reversibility with oleic acid at room temperature in toluene

In a 20 mL vial, 5 mL of 0.5 mg/mL colloidal solution of washed CGO-OAm-DT in toluene was stirred with 0.5 mL oleic acid for a week at room temperature. No visible optical change was observed. However, the UV-Vis spectra after a week showed a slight decrease in the 2.4-eV feature after 24 hours. This data indicates that the reverse ligand exchange is very slow at room temperature (Figure S12).

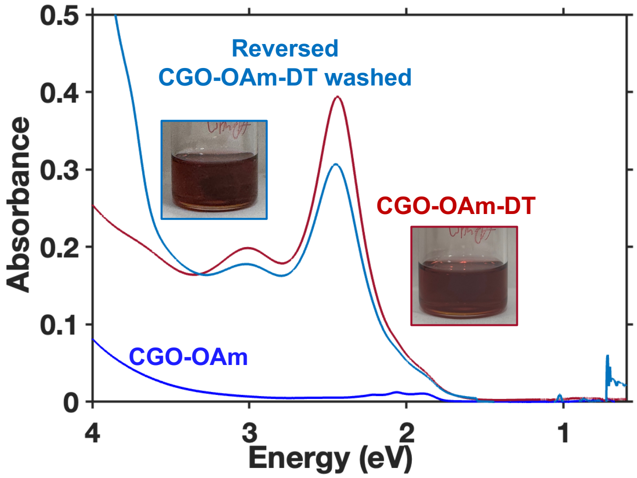

Figure S12. UV-Vis spectra of CGO-OAm (dark blue), CGO-OAm-DT (dark red), and CGO-OAm-DT after stirring with oleic acid for a week at room temperature (light blue). The absorption features at 2.4 eV and 3 eV are retained after oleic acid addition. Additional absorption observed at energies above 3.5 eV corresponds to oleic acid absorption.

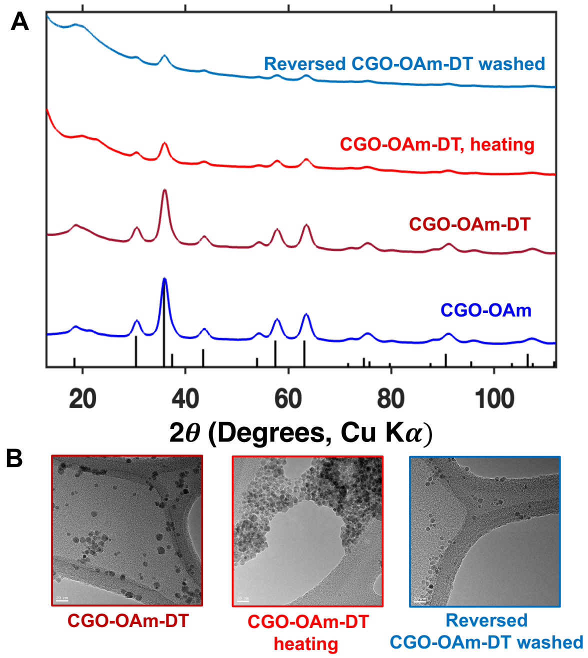

Figure S13. A) Powder XRD of CGO-OAm (dark blue), CGO-OAm-DT (dark red), and the product of the "reverse" ligand exchange reaction between CGO-OAm-DT and oleic acid in toluene (light blue), and the product obtained from heating CGO-OAm-DT in toluene in the absence of oleic acid (CGO-OAm-DT heating, light red) after 24 hours reaction. **B)** TEM images of CGO-OAm-DT, CGO-OAm-DT heating and reversed CGO-OAm-DT with oleic acid.

Reversibility with oleyl amine in toluene

In a 50 mL two-neck round bottom flask equipped with a water-cooled Liebig condenser and attached to a Schlenk line, 5 mL of 0.5 mg/mL colloidal solution of washed CGO-OAm-DT in toluene was degassed at room temperature with three cycles of evacuation followed by backfilling with nitrogen. The solution was continuously stirred and heated to reflux at 115 °C in a silicone oil bath. After about 30 minutes of heating, 0.5 mL of oleyl amine was added to the reaction. The red reaction mixture changed color to yellow-brown gradually after 24 hours (Figure S14).

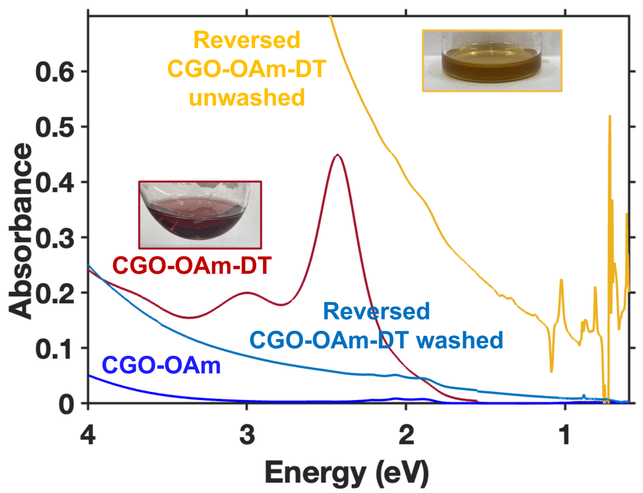

Figure S14. Reversibility with oleyl amine in toluene. UV-Vis spectra of CGO-OAm (dark blue), CGO-OAm-DT (dark red), and the product of the "reverse" ligand exchange reaction between CGO-OAm-DT and oleyl amine in toluene before washing (yellow), and after washing with methanol (light blue) after 24 hours reaction.

Reversibility in octadecene

To 5 mL of 0.5 mg/mL colloidal solution of washed CGO-OAm-DT in a 50-mL two-neck round bottom flask, 0.5 mL of oleic acid was added (Decanethiol: Oleic Acid ratio ~ 1:25, assuming negligible loss of nanocrystals after washing). The solution was degassed on a Schlenk line with three cycles of evacuation followed by backfilling with nitrogen at room temperature. The solution was continuously stirred and heated with a heating mantle connected to a temperature controller (Omega Engineering, Inc., Model CSC32K, Serial No. 17010355) programmed to a set point of 130°C. In the reversibility experiment with oleylamine, 0.5 mL of oleylamine was added instead of oleic acid. The red reaction mixture changed color gradually to clear blue as the temperature ramped to about 120 °C from room temperature within 10 minutes for the oleic acid reversibility experiment (Figure S15A) and to yellow for the oleylamine reversibility experiment. After centrifuging the yellow solution at 4000 rpm, a blue precipitate crashed out indicating the success of the reverse ligand exchange (Figure S15B). The experiment was repeated without the addition of oleic acid and oleyl amine, and a clear blue solution was still observed within 10 minutes, but the color change occurred when the temperature ramped to about 130 °C (Figure S15C).

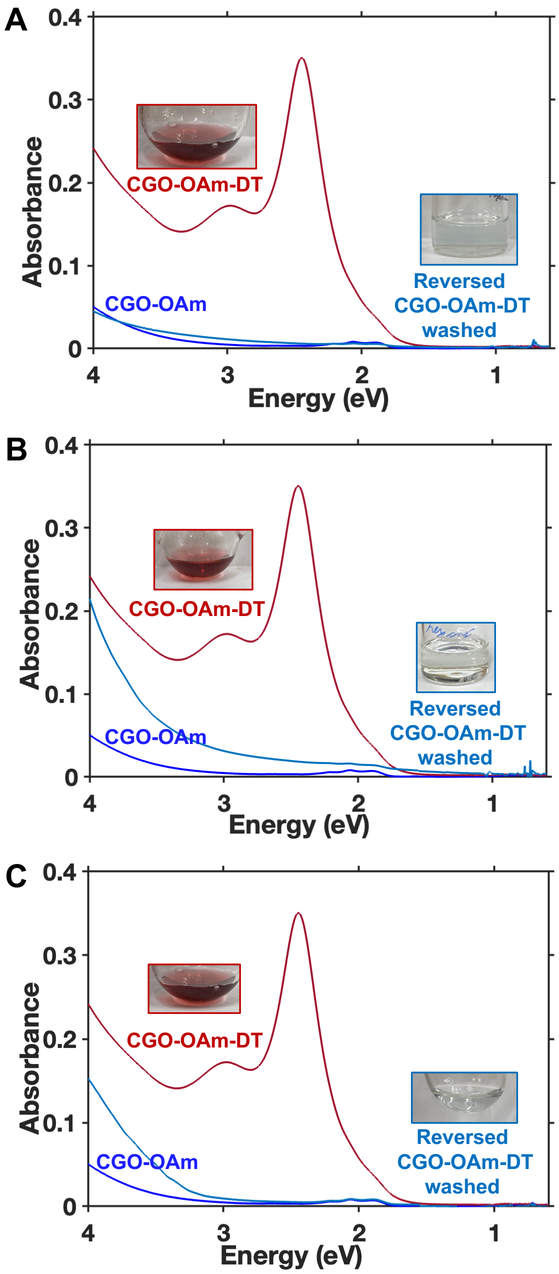

Figure S15. Reversibility in octadecene with the addition of oleic acid (A), oleyl amine (B), and without the addition of oleic and oleylamine (C). UV Vis spectra of CGO-OAm (before decanethiol treatment and reversibility reaction, dark blue), CGO-OAm-DT (after decanethiol treatment and before reversibility reaction, red), and reversed CGO-OAm-DT washed (after reversibility treatment with oleic acid (A), oleyl amine (B), and without the addition of oleic acid and oleyl amine (C), light blue).

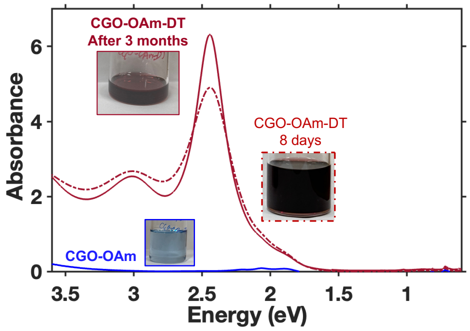

Figure S16. Colloidal dispersions of CGO-OAm (dark blue) and CGO-OAm-DT after 8 days (dotted lines), after 3 months (solid lines) in tetrachloroethylene.

Re-addition of thiols

The blue precipitate obtained after washing the CGO-OAm-DT-OA sample (CGO nanocrystals that were originally functionalized with OAm, treated with decanethiol, then heated in toluene in the presence of oleic acid) with methanol was redispersed in 5 mL of $CDCl_3$ and 774 µL of DT was added (approximately 1:2 ratio of OA:DT, considering negligible loss during washing). The solution turned to red gradually, and the optical features at 2.4 eV and 3 eV reappeared.

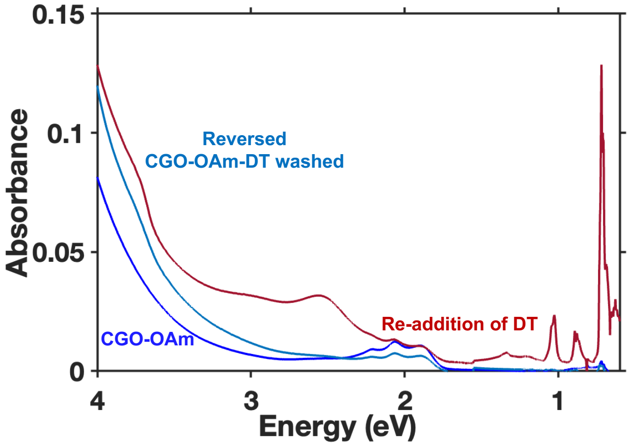

Figure S17. Re-addition of decanethiol to the reversed CGO-OAm-DT washed sample in $CDCl_3$ after a day. UV Vis spectra of CGO-OAm (before decanethiol treatment and reversibility reaction, dark blue), reversed CGO-OAm-DT washed (blue precipitate obtained after washing the CGO-OAm-DT-OA sample with methanol, light blue), and reversed CGO-OAm-DT washed after addition of decanethiol (red).

Ligand exchange reactions on molecular cobalt complexes

$Co(acac)_2$-DT and $Co(acac)_3$-DT: In a 20 mL vial, 67 μmol of cobalt (II) acetylacetonate ($Co(acac)_2$) or cobalt (III) acetylacetonate ($Co(acac)_3$) were dissolved in 2 mL of THF and then 55 μL DT (~1:4 Co:thiol ratio) was added. The $Co(acac)_2$ solutions changed color to reddish brown almost immediately while the $Co(acac)_3$ solution changed color after 2 days.

$CoFe_2O$ cluster-DT ligand exchange: $CoFe_2(\mu_3\text{-}O)(\mu_2\text{-}O_2CCF_3)_6(H_2O)_3$ was synthesized and purified according to our previous report.[9] In a 20 mL vial, 200 µL DT was added to 2 mL of a 10 mg/mL solution of this complex in acetone.

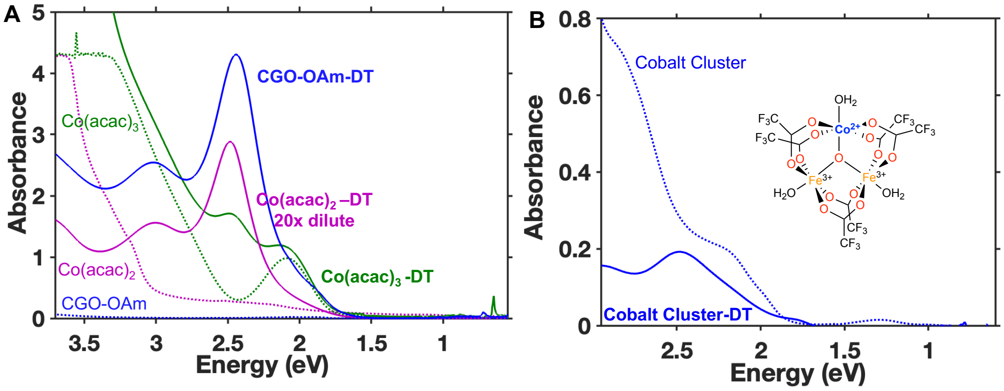

Figure S18. A) UV Vis spectra of cobalt (II) acetylacetonate, cobalt (III) acetylacetonate and CGO-OAm before and after decanethiol ligand addition (Spectra taken 2 days after the addition of DT). **B)** UV-Vis Spectra of cobalt cluster before and after the addition of DT.

REFERENCES

1. Springer-Verlag Berlin Heidelberg & Material Phases Data System (MPDS), Switzerland & National Institute for Materials Science (NIMS), Japan. https://materials.springer.com/isp/crystallographic/docs/sd_1201199
2. Higgs, T. C.; Ji, D.; Czernuszewicz, R. S.; Matzanke, B. F.; Schunemann, V.; Trautwein, A. X.; Helliwell, M.; Ramirez, W.; Carrano, C. J. The Fe (III), Co (III), and V (III) complexes of the "heteroscorpionate" ligand (2-thiophenyl) bis (pyrazolyl) methane. *Inorganic Chemistry* **1998**, *37*, 2383-2392.
3. Petroski, J.; Chou, M.; Creutz, C. The coordination chemistry of gold surfaces: Formation and far-infrared spectra of alkanethiolate-capped gold nanoparticles. *Journal of Organometallic Chemistry* **2009**, *694*, 1138-1143.
4. Loo, B. H.; Leahey, J.; Lee, Y. G. Surface chemistry of thiosemicarbazide on silver and copper electrodes. *Surface Science* **1990**, *226*, 119-130.

5. Loo, B. H.; Lee, Y. G.; Yazid, Z. Surface-enhanced Raman spectroscopic study of the metal—sulfur bonding: Adsorption of N,N-dimethyl-thioformamide on copper and silver electrodes. *Chemical Physics Letters* **1985**, *114*, 405-410.
6. Xie, B.; Numako, C.; Naka, T.; Takami, S. Color-controlled nonstoichiometric spinel-type cobalt gallate nanopigments prepared by supercritical hydrothermal synthesis. *Dalton Trans.* **2023**, *52*, 16285-16296.
7. Bourque, J. L.; Biesinger, M. C.; Baines, K. M. Chemical state determination of molecular gallium compounds using XPS. *Dalton Trans.* **2016**, *45*, 7678-7696.
8. Ghosh, S. C.; Biesinger, M. C.; LaPierre, R. R.; Kruse, P. X-ray photoelectron spectroscopic study of the formation of catalytic gold nanoparticles on ultraviolet-ozone oxidized GaAs(100) substrates. *Journal of Applied Physics* **2007**, *101*, 114322.
9. Sanchez-Lievanos, K. R.; Tariq, M.; Brennessel, W. W.; Knowles, K. E. Heterometallic trinuclear oxo-centered clusters as single-source precursors for synthesis of stoichiometric monodisperse transition metal ferrite nanocrystals. *Dalton Trans.* **2020**, *49*, 16348-16358.
